# *Rhodococcus* strains as source for ene-reductase activity

**DOI:** 10.1007/s00253-018-8984-7

**Published:** 2018-04-28

**Authors:** Bi-Shuang Chen, Rosario Médici, Michelle P. van der Helm, Ymke van Zwet, Lorina Gjonaj, Roelien van der Geest, Linda G. Otten, Ulf Hanefeld

**Affiliations:** 1 Biocatalysis, Department of Biotechnology, Delft University of Technology, Van der Maasweg 9, 2629 HZ Delft, The Netherlands; 2 Present Address: School of Marine Sciences, Sun Yat-Sen University, Guangzhou, 510275 People’s Republic of China; 3 Present Address: Department of Chemical Immunology, Leiden University Medical Center, Einthovenweg 20, 2333 ZC Leiden, The Netherlands

**Keywords:** *Rhodococcus*, Ene-reductase, Enantioselectivity, Asymmetric reduction

## Abstract

*Rhodococcus* strains are ubiquitous in nature and known to metabolise a wide variety of compounds. At the same time, asymmetric reduction of C=C bonds is important in the production of high-valued chiral building blocks. In order to evaluate if Rhodococci can be used for this task, we have probed several *Rhodococcus rhodochrous* and *R. erythropolis* strains for ene-reductase activity. A series of substrates including activated ketones, an aldehyde, an imide and nitro-compound were screened using whole cells of seven *Rhodococcus* strains. This revealed that whole cells of all *Rhodococcus* strains showed apparent (*S*)-selectivity towards ketoisophorone, while most other organisms show (*R*)-selectivity for this compound. Three putative ene-reductases from *R. rhodochrous* ATCC 17895 were heterologously expressed in *Escherichia coli.* One protein was purified and its biocatalytic and biochemical properties were characterised, showing typical (enantioselective) properties for class 3 ene-reductases of the old yellow enzyme family.

**Electronic supplementary material:**

The online version of this article (10.1007/s00253-018-8984-7) contains supplementary material, which is available to authorized users.

## Introduction

*Rhodococcus* strains are ubiquitous in nature, known to metabolise a wide variety of environmental pollutants (Bell et al. 1998; Martínková et al. 2009), including trichloroethene (Saeki et al. 1999), haloalkanes (Curragh et al. 1994) and dibenzothiophene (Monticello 2000). In addition to their value in bioremediation, *Rhodococcus* strains also show promise as biocatalysts in the synthesis of chiral compounds with their large number of enzymatic activities (Larkin et al. 2005). For example, most *Rhodococcus* strains harbour nitrile hydratases, a class of enzymes used in the industrial production of acrylamide and nicotinamide (Komeda et al. 1996). Some *Rhodococcus* strains are capable of transforming indene to 1, 2-indandiol, a key precursor of the anti-HIV drug Crixivan (Priefert et al. 2004). Many hydroxylating enzymes have been identified within bacteria belonging to the *Rhodococcus* strains, catalysing regio-, diastereo- and enantioselective hydroxylation of unactivated C–H bonds (O’Reilly et al. 2012). Besides these, many more biocatalytic activities could be discovered by using genome-based techniques (Ceniceros et al. 2017).

In another example, our group recently reported the Michael addition of water to a series of α,β-unsaturated carbonyl compounds in water with several *Rhodococcus* strains (Chen et al. 2015), which remains a major challenge for synthetic chemists (Resch and Hanefeld 2015). During these studies, the reduction of conjugated C=C bonds was also observed. The *Rhodococcus rhodochrous* ATCC 17895 cells reduced α,β-unsaturated cyclic ketones (2-cyclopentenone, 2-cyclohexenone and 2-cycloheptenone) into the corresponding saturated ketones as initially undesired side reaction for the addition of water to C=C bonds. 2-Cyclohexenone is a typical ene-reductase substrate (Scholtissek et al. 2017; Steinkellner et al. 2014), which is reduced to cyclohexanone, inferring the presence of an ene-reductase (ER), a class of enzymes that catalyse the reduction of activated C=C bonds. The asymmetric reduction of C=C bonds by chiral organometallic catalysts combined with molecular hydrogen has been extremely successful in the production of high-valued chiral building blocks (Simons et al. 2006). However, two limitations employing this strategy are the need to prepare complex ligands or use high hydrogen pressure. In contrast, ene-reductases from the Old Yellow Enzyme (OYE, EC 1.6.99.1) family are metal-independent flavoproteins, using a nicotinamide cofactor as hydride source and a conserved tyrosine residue for proton delivery (Stuermer et al. 2007). As a result of their broad applicability, ene-reductases received great interest in preparative organic synthesis. Indeed, since the very early discovery of the first member (OYE1) from *Saccharomyces pastorianus* (formerly known as *S. carlsbergensis*) in 1932 (Stuermer et al. 2007), many of them have been isolated from microorganisms and plants (Bougioukou and Stewart 2012; Stuermer et al. 2007; Toogood et al. 2012; Toogood and Scrutton 2014). Only recently, it was shown that a *Rhodococcus*, i.e. *R. opacus* CP1, contains multiple OYE-like proteins from which one was expressed and showed activity towards α,β-unsaturated carbonyl compounds (Riedel et al. 2015).

Herein, we describe several *R. rhodochrous* and *R. erythropolis* strains as sources for novel ene-reductases. We determined ene-reductase activity from seven wild-type *Rhodococcus* strains and found interesting (*S*)-selectivity for ketoisophorone in whole cell experiments. Three putative ene-reductases from *R. rhodochrous* ATCC 17895 were cloned and one was fully purified and characterised showing all characteristic ene-reductase properties.

## Materials and methods

### Materials and general methods

Vector pET-28a(+) was purchased from Novagen (Merck Millipore, Amsterdam, The Netherlands). Reduced form (NADH and NADPH) of nicotinamide adenine dinucleotide was from Sigma-Aldrich. All chemicals were purchased from Sigma-Aldrich (Schnelldorf, Germany) and were used without further purification unless otherwise specified. The culture media components were obtained from BD (Becton, Dickinson and Company, Breda, The Netherlands).

^1^H and ^13^C NMR spectra were recorded with Bruker Avance 400 (400 and 100 MHz, respectively) or Agilent 400-MR DD2 (400 MHz for ^1^H) instrument. Optical rotations were obtained at 20 °C with a Perkin-Elmer 241 polarimeter (sodium D line). Column chromatography was carried out with silica gel (0.060–0.200 mm, pore diameter ca. 6 nm) and with mixtures of petroleum ether (PE) and ethyl acetate (EtOAc) as solvents. Thin-layer chromatography (TLC) was performed on 0.20-mm silica gel 60-F plates. Organic solutions were concentrated under reduced pressure with a rotary evaporator.

Conversion of substrates and yield of products were quantified by GC using calibration lines with dodecane as an internal standard and the optical purity of the products were determined using chiral GC (Supporting Information section 5, Table S2 and Figs. S4–S14). The calibration lines were treated similarly as the reactions. The absolute configuration of 14, 15 and 19 was determined by comparing the results from the chiral GC analysis with those from a reaction with *Ts*OYE from *Thermus scotoductus* (Paul et al. 2013).

### General biotransformation procedure for substrate screening of *Rhodococcus* strains

Whole cells of *Rhodococcus* strains were obtained as described previously (Chen et al. 2015). Reactions were carried out in 1.5-mL screw-capped glass vials to prevent evaporation of substrate/product. Fifty milligrams of lyophilised cells was resuspended in 1 mL of potassium phosphate buffer (pH 7.0, 50 mM) and the substrate was added to a final concentration of 10 mM. The mixture was incubated at 30 °C in a thermoshaker (Eppendorf, Nijmegen, The Netherlands) for the given time (Table 1). Control experiments were performed in the absence of cells. For work-up, the cells were discarded after centrifugation at 13000 rpm for 2 min and the aqueous reaction mixtures (0.8 mL) were saturated with NaCl followed by extraction with ethyl acetate (containing internal standard) (2 × 0.4 mL). Combined organic layers were dried over Na_2_SO_4_ and measured on GC (Supporting Information section 5, Figs. S4–S14).

**Table 1:** Screening *Rhodococcus* strains for ene-reductase activity, measured by product formation

Substrate	*Rhodococcus* strains							Reaction time (h)
	A	B	C	D	E	F	G	
1a	++	++	++	+	++	++	+++	1
1b	+++	++	++++	++++	++++	++++	nd	2
1c	++	++	++	++	++	++	+++	1
2b	–	+	+	+	+	+	+	22
3	–	+	–	–	+	–	nd	2
4	+	+	+	+	+	+	+	1

Reaction conditions: 50 mg lyophilised cells in 1 mL 50 mM potassium phosphate buffer (pH 7.0), 10 mM substrate, 12.5 mM NADH. The mixture was incubated in a glass vial at 30 °C for the given amount of time. Yield defined as the amount of product produced: ++++ 60–100%; +++ 40–60%; ++ 20–40%; + 3–20%; − below 3%; nd not determined (%; determined by GC)

A = *R. rhodochrous* ATCC 17895; B = *R. erythropolis* DSM 43296; C = *R. erythropolis* DSM 43060; D = *R. erythropolis* NBRC 100887; E = *R. erythropolis* DSM 43066; F = *R. rhodochrous* DSM 43241; G = *R.* sp. R 312

### Molecular cloning of the ene-reductase genes and nucleotide sequence accession number

Three genes in the genome of *R. rhodochrous* ATCC 17895 were annotated as oxidoreductases from the OYE family (Chen et al. 2013). The sequences of these three putative ene-reductases *Rhr*ER 301, *Rhr*ER 2718 and *Rhr*ER 5439 were deposited at GenBank under accession numbers KT321319, KT321320 and KT321321, respectively. The genes were codon-optimised for *Escherichia coli* (deposited at GenBank under accession numbers *Rhr*ER 301: MG963278, *Rhr*ER 2718: MG963279 and *Rhr*ER 5439: MG963280), synthesised and cloned in frame with the *N*-terminal His-tag of expression vector pET28a(+) between the *Nde*I and *Hin*dIII restriction sites by BaseClear (Leiden, The Netherlands).

### Heterologous expression and purification of the ene-reductases

The recombinant plasmids were subsequently transformed into *E. coli* BL21 (DE3) cells. Expression was performed in LB medium containing 50 μg/mL kanamycin at 37 °C. When OD_600_ reached 0.5–0.6, the production of the recombinant ene-reductase was induced by addition of isopropyl thio-β-D-galactoside (IPTG) to a final concentration of 0.1 mM. For the determination of the optimal expression conditions, cultures were grown after induction between 17 and 37 °C and assayed after a period of 2 days. *E. coli* pET28a(+) empty was cultivated and induced with the same system as control experiment. Cells were harvested by centrifugation (10,000 rpm, 20 min, 4 °C) and washed two times with potassium phosphate buffer (pH 7.0, 50 mM). Harvested cells were resuspended in the same buffer and disrupted by sonication on ice (10 min, output 4, 40% duty cycle, Branson sonifier). After a centrifugation step (13,000 rpm, 30 min, 4 °C), the cleared crude extract was used for protein purification using a HisTrap affinity column (5 mL, GE Healthcare Life Sciences, Eindhoven, The Netherlands). The column was equilibrated with potassium phosphate buffer (pH 7.0, 50 mM). After loading the sample and elution of non-bound proteins with equilibration buffer, a linear gradient of imidazole (0–500 mM, 60 min) in potassium phosphate buffer was performed. The ene-reductase elutes at 130 mM imidazole. After a desalting step (PD-10 desalting column, Merck Millipore) with potassium phosphate buffer (pH 7.0, 50 mM), the enzyme was stored at − 20 °C prior to use.

### Protein analysis

Protein concentration was determined using the Bradford assay with BSA as a standard. SDS-PAGE was carried out on 4–12% Bis-Tris gels running in MOPS buffer using the Precision Plus Protein Standard as protein marker (Bio-Rad Laboratories, Veenendaal, The Netherlands).

### Activity assay

The ene-reductase activity was assayed by monitoring the oxidation of NADH through the decrease in UV at 340 nm using a molar absorption coefficient of 6.22 mM^−1^ cm^−1^. 2-Methyl-2-cyclopentenone 2a was used as standard substrate. One unit of activity was defined as the amount of enzyme catalysing the oxidation of 1 μmol NADH per minute under standard conditions (pH 7.0, 30 °C). The standard assay mixture (1 mL) was composed of 960 μL substrate solution (10 mM in potassium phosphate buffer (pH 7.0, 50 mM)), 20 μL NADH (12.5 mM in distilled water) and 20 μL enzyme solution (4 μM). Reactions were started by addition of the enzyme solution and measured for 1 min. The activity of the crude extract was determined using *E. coli* pET28a(+) under the same conditions.

### Determination of pH and temperature optima, stability and kinetic parameters

The optimum pH was determined by standard activity assay at different pH in the range of 5.0–11.0, with sodium citrate buffer (50 mM) for a pH range from 5.0–6.0, potassium phosphate buffer (50 mM) for a pH range from 6.0–9.0, glycine-NaOH buffer (50 mM) for pH 9.0–11.0. For the determination of the temperature optima, standard activity assay was performed at different temperatures in the range of 20–60 °C. The reaction mixtures were kept at each temperature for 5 min before NADH and enzyme solution was added to initiate the reaction. The activity at standard conditions (pH 7.0, 30 °C) was taken as 100%.

In order to determine its thermostability, the enzyme was incubated at different temperatures (20–70 °C) for 1 h, and the residual activity was measured using 2-methyl-2-cyclopentenone as the substrate at 30 °C by the standard activity assay. The activity of the enzyme without incubation at the given temperature was defined as 100%.

The kinetic parameters were determined by the standard activity assay with 2-methyl-2-cyclopentenone as substrate in duplicate at various concentrations (0.1–5 mM) and a constant NADH concentration (0.25 mM). The *K*_m_ and *k*_cat_ values were calculated from non-linear regression of Michaelis–Menten plots.

### Biotransformation of activated alkenes

Stock solutions of the substrates (100 mM), NADH (125 mM) and purified enzyme (238 μM) were prepared in potassium phosphate buffer (pH 7.0, 50 mM) with the exception of *N*-phenyl-2-methylmaleimide and 1-nitro-1-cyclohexene for which the 100 mM stock solutions were prepared in DMSO. The biotransformations were carried out in 1.5-mL screw-capped glass vials to prevent evaporation of substrate/product. Reactions were performed aerobically in a mixture (1.0 mL) containing potassium phosphate buffer (50 mM, pH 7.0), 10 mM of substrate, 12.5 mM of NADH and 4.8 μM of purified *Rhr*ER 2718. The reaction mixture was shaken for 4 h at 30 °C with 1000 rpm in a thermoshaker, cooled down on ice and extracted with 2 × 0.5 mL of ethyl acetate (containing 5 mM dodecane as internal standard). The combined organic layer was dried over Na_2_SO_4_ and measured with GC for conversion, yield and *ee* (Supporting Information, section 5, Figs. S4–S14).

### Preparative synthesis of 2-methyl-*N*-phenylsuccinimide

The reference compound 2-methyl-*N*-phenylsuccinimide was synthesised according to literature (Hall et al. 2007). *N*-phenyl-2-methylmaleimide (99 mg, 0.53 mmol) was dissolved in 10 mL of ethyl acetate. 10% Pd/C (11.4 mg) was used as catalyst for the 24 h hydrogenation at atmospheric pressure and room temperature with a hydrogen balloon. The solution was filtered over Celite and extracted with ethyl acetate. The extract was concentrated under reduced pressure, yielding 97.5% of racemic 2-methyl-*N*-phenylsuccinimide (96.5 mg, 0.51 mmol, 96% yield). ^1^H NMR (CDCl_3_) δ 1,47 (d, 3H, *J* = 7 Hz), 2.52 (dd, 1H, *J* = 17.4 Hz, *J* = 4 Hz), 3.01–3.10 (m, 1H), 3.11 (dd, 1H, *J* = 17.3 Hz, *J* = 9.2 Hz), 7.29–7.51 (m, 5H) in accordance with reported spectra (Supporting Information, section 7.1 and 7.2, Figs. S16 and S17) (Hall et al. 2007). The correct enantiomer of 2-methyl-*N*-phenylsuccinimide was determined using the ene-reductase isolated from *Thermus scotoductus* SA-01 (*Ts*OYE) (Opperman, Piater, and van Heerden 2008). *Ts*OYE produces > 99% (2*R*)-methyl-*N*-phenylsuccinimide using NADH as cofactor (Paul et al. 2013) (Supporting Information, section 5.8).

### Determination of the flavin species

An *Rhr*ER 2718 sample was incubated for 10 min at 99 °C to release the cofactor; denatured protein was removed by centrifugation at 14000 rpm for 30 min. The supernatant was spun through a Microcon YM3 (MWCO: 3000 Da, Merck Millipore) centrifugal concentrator device (14,000 rpm, 30 min) to remove residual protein. The resulting sample was analysed by high-performance liquid chromatography (HPLC) to identify the flavin cofactor. For the separation and quantification of flavin adenine dinucleotide (FAD) and flavin mononucleotide (FMN), a reverse phase C18 HPLC column connected to a Shimadzu LC10Ai HPLC system was used (Shimadzu Benelux, ‘s-Hertogenbosch, The Netherlands). Ammonium acetate (50 mM, pH 6.0) and 70% acetonitrile in ammonium acetate (50 mM, pH 6.0) were used as mobile phase. The retention times of FAD and FMN are 6.53 and 9.12 min, respectively (Supporting Information, section 4).

### Preparative synthesis of 2-methylcyclopentanone 14 catalysed by RhrER2718 expressed in *E. coli*

For isolation and characterisation of the reduction product of 2-methyl-2-cyclopentenone 2a, the reaction was carried out on preparative scale. Two hundred fifty milligrams of the lyophilized cells of *E. coli* expressing *Rhr*ER 2718 was resuspended in 50 mL of potassium phosphate buffer (50 mM, pH 7.0), and substrate 2-methyl-2-cyclopentenone (2a; 98 mg, 1 mmol) and NADH (1 mmol) was added. Reaction was incubated at 30 °C and shaken at 180 rpm for 4 h. Then, the cells were removed by centrifugation and the supernatant was saturated with NaCl and then extracted with ethyl acetate. The extract was concentrated under reduced pressure and purified by flash column chromatography on silica gel (eluent: PE/EtOAc 1:1) to yield 2-methylcyclopentanone (14; 80 mg, 0.80 mmol, 80% yield, 86% *ee*) as a colourless oil; GC-MS and GC analysis were identical with the data of the commercial sample confirming that the reduction occurred at the C=C bond; m/z: 98 (M^+^, 33), 83 (9), 80 (3), 70 (18), 69 (30), 56 (16), 55 (51), 54 (4), 53 (4), 43 (13), 42 (100), 41 (43), 40 (7) (Supporting Information, section 6, Fig. S15).

### Preparative synthesis of (S)-levodione catalysed by *R. rhodochrous* ATCC 17895

Cell pellets collected from 3 L medium were used to prepare the cell-free extract by a French press (2.05 kBar, 2 shots) with 300 mL of potassium phosphate buffer (50 mM, pH 7.0), and substrate ketoisophorone (4; 450 mg, 2.96 mmol) was added. The reaction was incubated at 30 °C and shaken at 180 rpm for 1 h. The reaction mixture was saturated with NaCl and then extracted with ethyl acetate. The extract was concentrated under reduced pressure and purified by flash column chromatography on silica gel (eluent: PE/EtOAc 5:1) to yield (*S*)-levodione (18; 137 mg, 0.88 mmol, 30% yield, 75% *ee*) as a colourless oil; [α]_D_^20^ = + 261° (*c* 0.34, methanol) (literature [α]_D_^20^ = + 270° (*c* 0.2, methanol) for (*S*)-levodione (Leuenberger et al. 1976)); ^1^H NMR (400 MHz, CDCl_3_): δ: 1.09 (s, 3H), 1.12 (d, *J* = 6.8 Hz, 3H), 1.19 (s, 3H), 2.32 (dd, *J* = 13.2 Hz, 17.6 Hz, 1H), 2.50 (dd, *J* = 15.2 Hz, 0.7 Hz, 1H), 2.71–2.75 (m, 2H), 2.95–3.02 (m, 1H); ^13^C NMR (100 MHz, CDCl_3_) δ: 14.5, 25.6, 26.5, 39.8, 44.2, 44.8, 52.7, 207.9, 214.0 ppm (in accordance with literature (Fryszkowska et al. 2009), Supporting Information, section 5.12, 7.3 and 7.4, Figs. S18 and S19).

## Results

### Ene-reductase activity from wild-type *Rhodococcus* strains

Ene-reductase activity has been found in many different organisms, from bacteria through fungi and plants (Faber and Hall 2015; Scholtissek et al. 2017). Although many other enzymatic activities (nitrile hydratase, Michael hydratase, hydroxylating enzyme, mono- and dioxygenases) have been identified in *Rhodococcus* strains, only recently the first ene-reductase has been described in *Rhodococcus opacus* 1CP (Riedel et al. 2015). Members of the ene-reductase family typically catalyse the reduction of C=C bonds conjugated to carbonyl and heterocarbonyl groups (Stuermer et al. 2007). We started our investigation by screening a series of seven typical ene-reductase substrates using whole cells of 7 different *Rhodococcus* strains. We chose a range of α,β-unsaturated cyclic ketones with different ring sizes and substitution patterns (1, 2), one cyclic nitro-alkene (3) and the standard ene-reductase substrate ketoisophorone (4). Based on phylogenetic analysis as described previously (Chen et al. 2013), we decided to investigate *R. rhodochrous* ATCC 17895, 5 closely related and one unrelated *Rhodococcus* strain: *R. erythropolis* DSM 43296, *R. erythropolis* DSM 43060, *R. erythropolis* NBRC 100887, *R. erythropolis* DSM 43066, *R. rhodochrous* DSM 43241, *R.* sp. R312. The screening was performed on a 1-mL scale with 10 mM of substrate and 50 mg lyophilised cells of the corresponding organism. As shown in Table 1, all of the seven organisms catalysed the reduction of activated C=C bonds of a variety of typical ene-reductase substrates with slightly different activity. These variations might be due to the fact that different strains display different expression of enzymatic activities, although all are from the same genus. This is also seen in other organisms, for example the characterised ene-reductase “xenobiotic reductase (XenA)” from *Pseudomonas putida* II-B was reported to reduce 2-cyclohexenone with the activity of 4.1 U/mg and 2,4,6-trinitrotoluene with the activity of 0.5 U/mg, while the 51% similar ene-reductase from *P. fluorescens* I-C (XenB: xenobiotic reductase B) converted these substrates with the activity of 0.6 and 2.5 U/mg, respectively (Blehert et al. 1999).

**Fig. 1:**
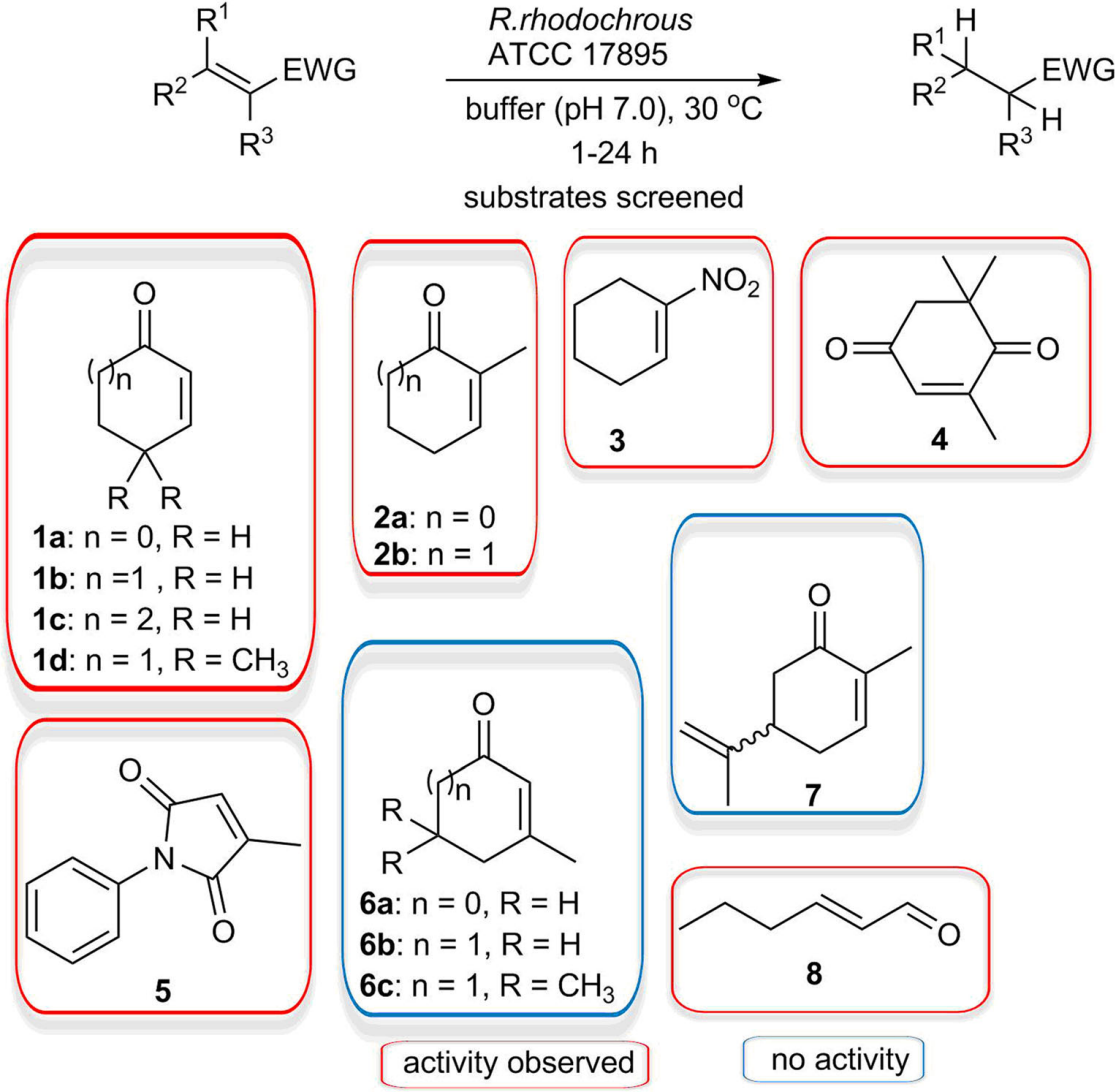
Substrate screening for ene-reductase activity of *R. rhodochrous* ATCC 17895. Activity is based on product yield: substrates in red boxes were converted to the expected product and blue boxes indicate no conversion at all or conversion to unwanted products

As we have sequenced the genome of *R. rhodochrous* ATCC 17895 (Chen et al. 2013), we continued screening eight more substrates with this strain, resulting in a series of 14 typical ene-reductase substrates (Fig. 1). We expanded the range of α,β-unsaturated cyclic ketones with different ring sizes and substitution patterns (1d, 2a, 6, 7) and added the well-known OYE substrate 2-methyl-*N*-phenylmaleimide (5) and an α,β-unsaturated aldehyde (8) as example of a non-cyclic substrate. The results are summarised in Fig. 1 (red: reasonable to high activity; blue: low to no activity). It should be mentioned here that for each of these biotransformations, we studied the conversion of substrates based on the consumption of substrates and yield of products related to the formation of the desired product, and found significant mass balance problems. Due to the different evaporation rate of alkene and saturated compound, the yield of the rather volatile ketone products are notoriously underestimated. Furthermore, whole cells have many other enzymes present, resulting in the formation of many by-products. In general, 70% of all substrates was at least converted, but from Table 1, it is clear that for most products, < 40% was recovered, either due to extraction problems or the activity of other enzymes in the cell.

As shown in Fig. 1 and Table 1, all *Rhodococci* showed varying ene-reductase activity towards the selected substrates (analysis conditions see Table S2 and Figs. S4–S14). In general, six-membered rings are the best substrates, while activity decreases with more bulky substrates. 2-Methyl-2-cyclopentenone (2a) was only probed with *R. rhodochrous* ATCC 17895 cells. It was converted the fastest (99% conversion within 1 h) resulting in good product formation, but unfortunately both enantiomers were produced equally. For α,β-unsaturated cyclic ketones, substituents on the ring greatly affect the ene-reductase activity. Similar to other known ene-reductases, the α-position methylated α,β-unsaturated cyclic ketones were converted (2 and 4) by all *Rhodococcus* ene-reductases. Other substrates were converted slower and the conversion stopped at lower yields. From the substrates that were only screened with *R. rhodochrous* ATCC 17895 cells, none of 3-methylcyclopente-1-one (6a), 3-methyl 2-cyclohexenone (6b) or isophorone (6c) were converted into the desired product, implying that *R. rhodochrous* ATCC 17895 does not have ene-reductases that can accept β-methylated ketones. This last hypothesis is also supported by the fact that (*R*)-(−) or (*S*)-(+)-carvone (7) were not a substrate, even though there is a methyl group in the α-position. The acyclic aldehyde *trans*-2-hexenal (8) was reduced within 1 h into the corresponding saturated aldehyde and then further to the corresponding alcohol. This additional carbonyl reduction can be contributed to the use of whole cell as other enzyme activities are also present, like alcohol dehydrogenases. All these results revealed that the ene-reductase activity is not limited to one *Rhodococcus* strain, but may be a general feature in Rhodococci, which greatly expands the biocatalytic toolbox for the reduction of activated C=C bonds.

The stereoselectivity of the *Rhodococcus* ene-reductases was assessed with the reduction product of ketoisophorone (4). As presented in Table 2, all seven tested *Rhodococcus* strains gave apparent (*S*)-selectivity to ketoisophorone with 55–95% *ee* (analysed by chiral GC, see Supporting Information section 5), except for *R. erythropolis* DSM 43296 (strain B), which showed low (*R*)-selectivity. It is worth noting that the biochemical procedure for the preparation of (*R*)-levodione from ketoisophorone by biocatalytic reduction with OYEs is known. Indeed, all the reported ene-reductases from the OYE family which are found in plants, yeasts, bacteria and parasites only show (*R*)-selectivity to ketoisophorone. In other words, there are many biocatalysts employing ene-reductases related to the production of enantiomerically pure (*R*)-levodione, while access to the biocatalytic production of (*S*)-levodione is still missing. So far, (*S*)-levodione was chemically made by a very complex synthesis (Leuenberger et al. 1976) using toxic chromium trioxide. Biocatalytically, until recently, only two enzymes *Ph*ENR from *Pyrococcus horikoshii* and *Tt*ENR from *Thermus thermophilus* were described (Steinkellner et al. 2014) to reduce ketoisophorone to (*S*)-levodione with 28% conversion and 87% *ee*, 8% conversion and 14% *ee*, respectively, as also shown in Table 2. Both of them were originally annotated as putative styrene monooxygenase, but were renamed as NADPH-dependent quinone reductase based on their catalytic activity. They have, however, a different protein size, folding type and low sequence identity to OYEs although they do share similar arrangements of active site functional groups. In other words, they are not belonging to the OYE family while they have promiscuous ene-reductase activity and they show approximate mirror symmetry to the ene-reductase active site.

**Table 2:** Reduction of ketoisophorone 4 catalysed by whole cells of different *Rhodococcus* strains, and other enzymes from literature

Catalyst	Yield of levodione 18 (%)	*ee* of levodione (%)	Reference
Strain A	7	75 (*S*)	This study
Strain B	9	10 (*R*)	This study
Strain C	7	68 (*S*)	This study
Strain D	7	81 (*S*)	This study
Strain E	9	91 (*S*)	This study
Strain F	7	54 (*S*)	This study
Strain G	12	93 (*S*)	This study
*Ph*ENR	28	87 (*S*)	(Steinkellner et al. 2014)
*Tt*ENR	8	14 (*S*)	(Steinkellner et al. 2014)

Reaction conditions: 50 mg lyophilised cells in 1 mL 50 mM potassium phosphate buffer (pH 7.0), 10 mM substrate, 12.5 mM NADH; incubation at 30 °C for 1 h

A = *R. rhodochrous* ATCC 17895; B = *R. erythropolis* DSM 43296; C = *R. erythropolis* DSM 43060; D = *R. erythropolis* NBRC 100887; E = *R. erythropolis* DSM 43066; F = *R. rhodochrous* DSM 43241; G = *R.* sp. R 312; *Ph*ENR = *Pyrococcus horikoshii*; *Tt*ENR = *Thermus thermophilus*

Six *Rhodococcus* strains reduced ketoisophorone to (*S*)-levodione with low yield and high enantioselectivity (no improvement was achieved by increasing the reaction time or doubling the amount of cells). Similar results were obtained with the purified (*S*)-selective enzymes described earlier (Table 2). To obtain higher yields of (*S*)-levodione, reactions were performed with whole cells of *R. rhodochrous* ATCC 17895 with the addition of NADH and NADPH in parallel since ene-reductase in general uses a nicotinamide cofactor as hydride source (Bougioukou and Stewart 2012). No acceleration compared to the original reaction without cofactor addition was observed (data not shown), implying that the whole-cell system can generate enough NAD(P)H for the reduction by cellular metabolism or the enzyme is not NAD(P)H dependent. Notably, when the reaction was performed with the cell-free extract of *R. rhodochrous* ATCC 17895, the yield of (*S*)-levodione was increased from 18% within 1 h to 40% after 7 h, without significant change of enantioselectivity. In preparative scale, ketoisophorone (450 mg, 3.26 mmol) was converted to (*S*)-levodione (137 mg, 0.98 mmol), which was obtained with 30% isolated yield and 75% *ee* using the cell-free crude extract of *R. rhodochrous* ATCC 17895. The absolute configuration was further confirmed as (*S*) via measurement of the optical rotation from the purified product: [α]_D_^20^ = + 261° (*c* = 0.34, methanol), and comparison with literature data [α]_D_^20^ = + 270° (*c* = 0.4, methanol) (Leuenberger et al. 1976).

In order to evaluate whether the (*S*)-levodione was produced by an enantioselective ene-reductase, or both enantiomers were produced and the (*R*)-levodione was consumed by another enzyme the reversible reaction was undertaken. The reaction was performed under aerobic condition with (*R*)- or (*S*)-levodione as a substrate using crude cell extract of *R. rhodochrous* ATCC 17895 with added NADH or NAD^+^. In all reactions, the levodione was not converted. Some racemisation was observed, which also happened during incubation in potassium phosphate buffer (pH 7.0) only and had been reported before (Fryszkowska et al. 2009). Therefore, there seem to be no other enzymes in *R. rhodochrous* ATCC 17895 that can catalyse this conversion under these conditions. However, the dehydrogenation of saturated ketones by OYEs has been shown to occur in vitro, but only at higher temperatures (Schittmayer et al. 2011) or by changing the redox potential of the flavin cofactor (Murthy et al. 1999) to overcome the high activation energy barrier, explaining the lack of product in this experiment. As *R. rhodochrous* ATCC 17895 is a mesophilic organism, the corresponding reaction could not be performed. Overall, this indicates that there is no (*S*)-selective ene-reductase present.

### Isolation and characterisation of ene-reductases from *R. rhodochrous* ATCC 17895

#### Annotation of ene-reductases

In order to identify the ene-reductases in *Rhodococcus* strains, bioinformatics studies were performed. In previous work, we have sequenced and annotated *R. rhodochrous* ATCC 17895 (Chen et al. 2013) and three candidates were suggested as ‘OYE family NADH flavin oxidoreductase’. The predicted amino acid sequences were subsequently designated as *Rhr*ER 301, *Rhr*ER 2718 and *Rhr*ER 5439. A multiple sequence alignment was performed with 2–3 sequences from each class of known ene-reductases (Scholtissek et al. 2017) using the online available Clustal Omega alignment tool (Sievers et al. 2011). As shown in the supplemental information (Fig. S1), considerable sequence identities were observed for all three putative ene-reductases (from 20 to 55%) over the entire sequence with respect to known OYEs.

**Fig. 2:**
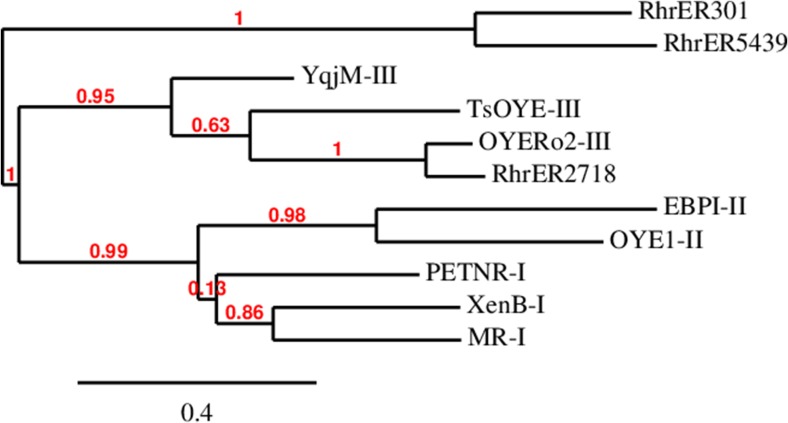
Phylogenetic relationship of *Rhr*ERs to other OYEs with known function from different classes (Scholtissek et al. 2017). The tree was constructed using the “One-Click” Mode on Phylogeny.fr (Dereeper et al. 2008), using the same OYEs as in the sequence alignment (Fig. S1). The class of known OYEs is written behind the name. Branch support values are indicated in red

From the phylogenetic tree of the same data (Fig. 2), it is clear that *Rhr*ER2718 is closest to another rhocococcal ene-reductase, and all new genes cluster in or close to class 3 OYE. This is also confirmed on the specific amino acid level. *Rhr*ER 2718 exhibits all six residues for substrate binding in the active site, while five amino acids out of six from *Rhr*ER 301 and *Rhr*ER 5439 are identical with class 3 OYEs (Fig. S1, the black frames indicate the substrate binding sites). Interestingly, the different amino acid in *Rhr*ER 301 and *Rhr*ER 5439 is also not similar to an amino acid of one of the other classes. It is noteworthy that these 2 enzymes seem to have an extended loop between α-helix 6 and β-sheet 6, which could all point towards a different substrate specificity of these enzymes.

Furthermore, it is known that the catalytic site of ene-reductases harbours a flavin cofactor, which donates a hydride onto the β-carbon of the substrate, which is usually a flavin mononucleotide (FMN) in class 3 enzymes. Several highly conserved FMN binding sites are present in all the three putative ene-reductases which are shown by grey shading (Fig. S1). In addition, a pair of amino acid residues (typically histidine/histidine or asparagine/histidine) located in the enzyme binding pocket, that act as H-bonding donors to the electron-withdrawing group of the substrate, and a conserved tyrosine residue, that is necessary to deliver a proton onto the α-carbon of the substrate, are also present in all three putative *Rhodococcus* ene-reductases (white letters on black background, Fig. S1).

#### Cloning, protein expression, purification and characterisation of putative ene-reductases

The three putative ene-reductases were ordered codon-optimised for *E. coli* and cloned into pET28 for heterologous expression in *E. coli* with *N*-terminal His_6_-tag. Unfortunately, only *Rhr*ER 2718 is predominantly present in the soluble fraction (Supporting Information, Fig. S2). The expression of *Rhr*ER 301 and *Rhr*ER 5439 resulted in no expression at all, or only insoluble expression, even after several attempts to improve this (Supplementary Information, section 2, Table S1). As the analysis of the gene already showed some deviations from other OYEs, we decided not to pursue the expression of these enzymes any further.

**Fig. 3:**
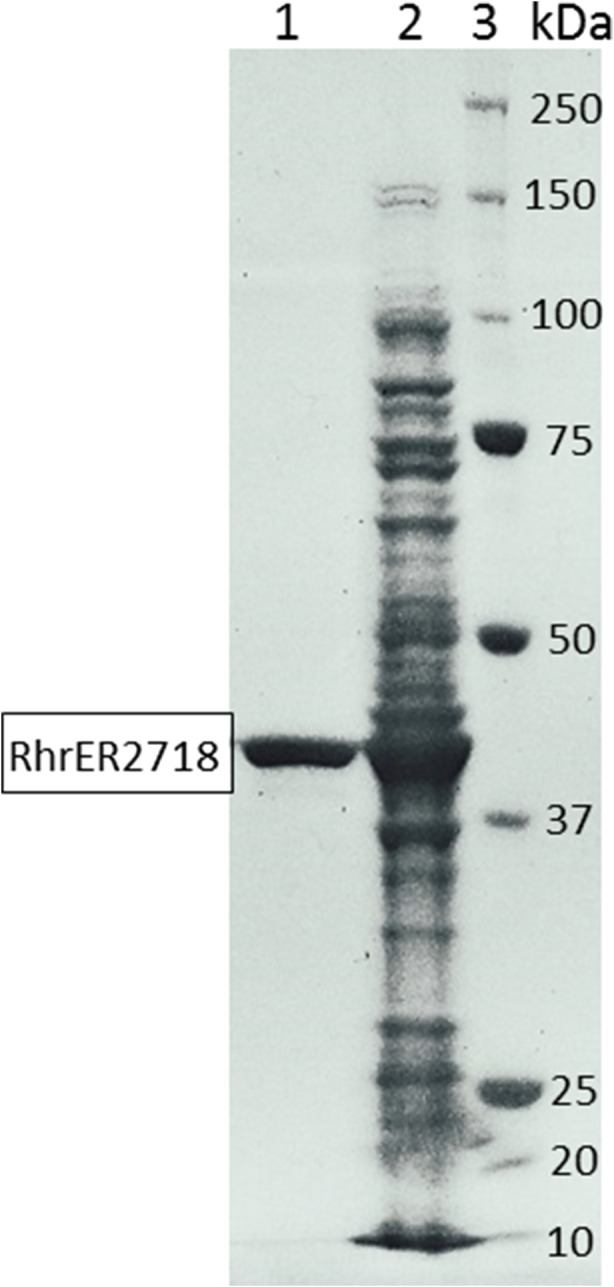
SDS-PAGE gel analysis of the purified *Rhr*ER 2718 from *R. rhodochrous* ATCC 17895. Lane 1, purified ene-reductase; lane 2, crude extract from *E. coli* expressing the gene; lane 3, molecular weight marker (BioRad Precision Plus Protein Standard)

*Rhr*ER 2718 was purified by a single step using nickel affinity chromatography and this preparation was used for all further investigations. The resulting specific activity of pure *Rhr*ER 2718 on 2-methyl-2-cyclopentenone 2a was 0.92 U/mg. SDS-PAGE analysis revealed that a single band with an apparent molecular size of 40 kDa (Fig. 3, lane 1), corresponds to the enzyme, confirming the theoretically calculated molecular mass (39.84 kDa) derived from the amino acid sequence. The native molecular weight was determined to be 75 kDa by native gel electrophoresis (PAGE), which suggests a dimeric structure for *Rhr*ER 2718, in line with other enzymes from class 3, like *Ts*OYE from *Thermus scotoductus* (Opperman et al. 2008).

Studies concerning cofactor dependency of the ene-reductase *Rhr*ER2718 revealed that the cofactor NADH is highly preferred, and almost no activity was detected when NADPH was tested as cofactor with 2-methyl-2-cyclopentenone as a substrate. As such, *Rhr*ER 2718 is an NADH-dependent ene-reductase. In addition, the coenzyme requirement of the ene-reductase *Rhr*ER 2718 was investigated. The conserved catalytic tyrosine (Tyr186) can be clearly seen and the residues for substrate binding and FMN binding are also present (Fig. S1). These results highly suggest that FMN is the coenzyme of *Rhr*ER 2718. This was confirmed by the observation of FMN in the HPLC analysis (Supporting Information Fig. S3) of the denatured enzyme solution.

#### Effect of pH and temperature, thermostability, and kinetic parameters

**Fig. 4:**
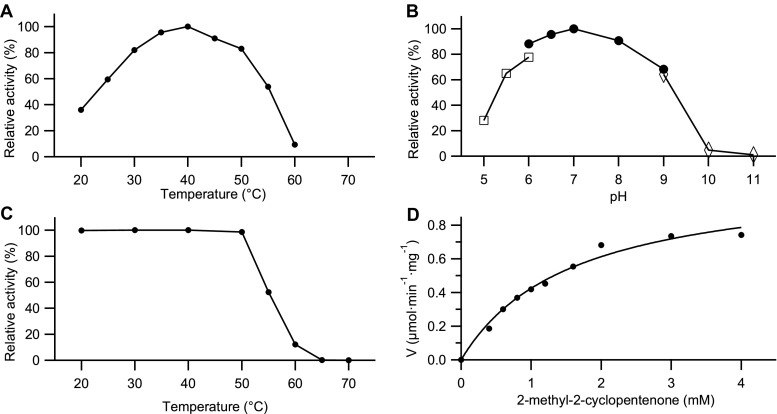
Temperature optima (**a**), pH optima (**b**), thermostability (**c**) and Michaelis–Menten kinetics (**d**) of the purified *Rhr*ER 2718. The activity was measured using the standard UV assay towards 2-methyl-2-cyclopentenone 2a. **a** All the reaction mixtures were kept at given temperatures for 5 min before NADH and enzyme solution were added to initiate the reaction. **b** The activity was assayed in the following 50 mM buffers: (i) sodium citrate (pH 5.0–6.0) (□), (ii) potassium phosphate (pH 6.0–9.0) (●), and (iii) glycine-NaOH (pH 9.0–11.0) (◊); reaction mixtures were incubated at 30 °C for 5 min before NADH and enzyme solution were added to initiate the reaction. **c** The enzyme solutions were kept for 1 h at each temperature before the samples were withdrawn to measure the residual activity in the soluble protein content. **d** *V*_max_ = 1.1 μmol∙min^−1^∙mg^−1^ and *K*_m_ = 1.6 mM

The influence of temperature and pH on the enzyme activity and stability were investigated by monitoring the change in enzyme activity towards 2-methyl-2-cyclopentenone 2a (Fig. 4). The temperature profile of the purified *Rhr*ER 2718 revealed that the enzyme exhibited an optimal activity at 40 °C, while it steeply decreased over 50 °C, with only 10% relative activity at 60 °C (Fig. 4a). The activity-pH profile of the enzyme gave a broad peak over the range of pH 5.0–9.0. However, the enzyme showed no detectable activity at pH 10.0 or higher. The optimal activity was observed at pH 7.0 in potassium phosphate buffer (Fig. 4b). Studies concerning thermostability were performed at different temperatures from 20 to 70 °C (Fig. 4c). The results show that the purified *Rhr*ER 2718 is relatively stable under 50 °C, but lost its activity almost completely after incubation at 60 °C for 1 h. Moreover, the enzyme is stable at 30 °C for 24 h without any activity loss (data not shown). For 2-methyl-2-cyclopentenone, the Michaelis–Menten plot (Fig. 4d) allowed calculation of the affinity constant *K*_m_ to be 1.6 mM and the maximal specific activity (*V*_max_) to be 1.1 μmol min^−1^ mg^−1^.

#### Biotransformation with purified RhrER 2718

In order to assess the substrate scope and the stereospecificity of *Rhr*ER 2718, we screened the same substrates which were accepted by whole cells of *R. rhodochrous* ATCC 17895 (Fig. 1, Table 3). As expected, *Rhr*ER 2718 catalysed the reduction of cyclic and linear alkenes conjugated with carbonyl or nitro groups, although some deviations from the trends with whole cells of *R. rhodochrous* ATCC 17895 were observed. The isolated enzyme displays lower yields with cyclic ketones having larger rings, and methyl groups on the α-carbon reduce the yields even further. Substituents on other positions of the ring seem to have various effects, which show a different trend when compared to the whole cell catalysis. These effects are in agreement with other ene-reductases of the OYE family (Toogood et al. 2010). The relative low yields in comparison to reported yields in other enzymes (Scholtissek et al. 2017) can be explained by the fact that our reactions were done under aerobic conditions, thereby oxidising the NADH present (Toogood et al. 2012). Another explanation could be the poor stability of the enzyme while shaking relatively fast, as often a white precipitate was observed at the end of the reactions.

**Table 3:**
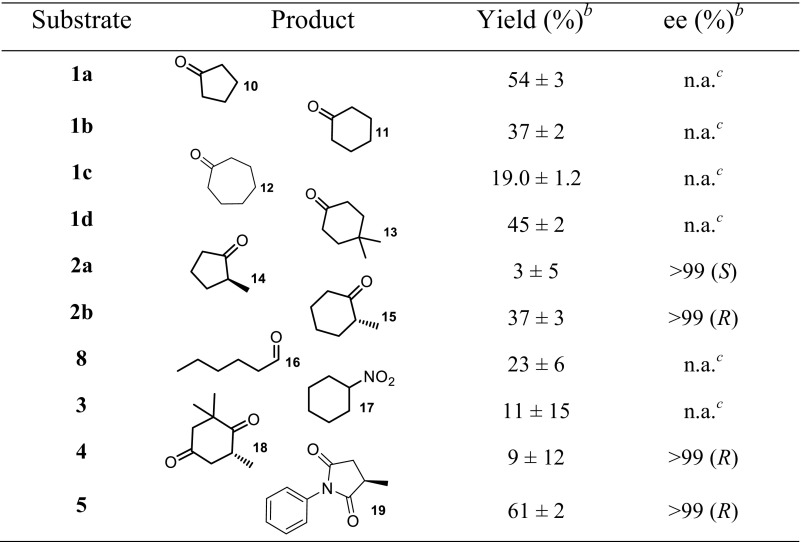
Substrate scope for the reductions of α,β-unsaturated carbonyl compounds (1–8) by pure *Rhr*ER 2718^a^

^a^Conditions: 50 mM potassium phosphate buffer (pH 7.0), [substrate] = 10 mM, [NADH] = 12.5 mM, [*Rhr*ER 2718] = 200 μg/mL, temp. = 30 °C, reaction time = 4 h, 1000 rpm

^b^Product formation determined by GC analysis as percentage of starting substrate concentration

^c^n.a. = not applicable

## Discussion

There are two large discrepancies between conversions with whole cells and pure enzyme in this research. Whole cells of *R. rhodochrous* ATCC 17895 produce (*S*)-levodione from ketoisophorone, while the enzyme *Rhr*ER 2718 is (*R*)-selective. Although the results are not conclusive we propose that the whole cells produce a mixture of (*S*)- and (*R*)-levodione, while an (*R*)-selective enzyme converts the (*R*)-levodione further leaving (*S*)-levodione behind. This results in an apparent (*S*)-selectivity for the whole cells, although the purified ene-reductase has the opposite selectivity.

The second dissimilarity lies in the reduction of 2-methyl-2-cyclopentenone (2a), which shows the highest activity using whole cells, while yielding racemic product, suggesting that more ene-reductases are able to convert this substrate or racemases are present in the whole cells. In contrast, the yield with pure enzyme is only 3% after 4 h, but producing 99% (*S*)-2-methylcyclopentanone. This is particularly surprising in view of the fact that 2a is the substrate of the activity assay and no inhibitory effects were observed in the kinetic studies (Fig. 4). Therefore, the reaction was scaled with a significant excess of the enzyme *Rhr*ER 2718. This time 80% yield albeit with 86% ee were observed. This indicated that indeed enzyme instability under reaction conditions might be the limiting factor in the substrate screening (Table 3). It is worth mentioning that the inexplicable change of enantioselectivity for formation of (*S*)-2-methylcyclopentanone 14, while all other biotransformations of similar substrates result in (*R*)-conformations, has also been reported for all other OYEs. Although no satisfying explanation has been reported yet (Fryszkowska et al. 2009), work from the group of Stewart might infer that substrate 2a could bind in a ‘flipped’ orientation (Padhi et al. 2009), while maintaining the optimal Cβ-FMN N5 distance and angle (105°) (Fraaije and Mattevi 2000).

In summary, ene-reductase activity was discovered in different *Rhodococcus* strains. The enzyme-catalysed asymmetric C=C reduction of a series of activated alkenes as substrates were shown. From three OYE candidates, only the encoded protein *Rhr*ER 2718 could be expressed, purified and characterised, showing typical ene-reductase properties from class 3 OYEs (Scholtissek et al. 2017). This enzyme was identified as an NADH-dependent ene-reductase, thus belonging to the few family members of ene-reductases with a preference for NADH.

## Electronic supplementary material

ESM 1 (PDF 4420 kb)
