# Supplementary material for: Rhodococcus strains as source for ene-reductase activity
Source: Appl Microbiol Biotechnol. 2018 Apr 28;102(13):5545–56. doi: 10.1007/s00253-018-8984-7 (PMC5999131; doi:10.1007/s00253-018-8984-7)
Supplement: Supplementary file 1 — (PDF 4420 kb) [file 253_2018_8984_MOESM1_ESM.pdf]

Electronic Supplementary Material for:

Applied Microbiology and Biotechnology

Rhodococcus strains as source for ene-reductase activity

Bi-Shuang Chen[1,2,#], Rosario Médici[1,#], Michelle P. van der Helm[1], Ymke van Zwet[1], Lorina Gjonaj[1,3], Roelien van der Geest[1], Linda G. Otten[1], Ulf Hanefeld[1,*]

[1] *Biocatalysis, Department of Biotechnology, Delft University of Technology, Van der Maasweg 9, 2629 HZ Delft, The Netherlands*

[2] *Current address: School of Marine Sciences, Sun Yat-Sen University, Guangzhou 510275, People's Republic of China*

[3] *Current address: Department of Chemical Immunology, Leiden University Medical Center, Einthovenweg 20, 2333 ZC Leiden, The Netherlands.*

[#] These authors contributed equally to this paper

*Corresponding Author:

U. Hanefeld, Tel: +31(0)15-2789304; Fax: +31(0)15-2781415; Email: u.hanefeld@tudelft.nl.

Biocatalysis, Department of Biotechnology, Delft University of Technology, Van der Maasweg 9, 2629 HZ Delft, The Netherlands

Table of contents

1. Sequence alignment of the three putative ene-reductases from *R. rhodochrous* ATCC 17895 with selected ene-reductases from all 3 OYE classes
2. Solubilisation of *Rhr*ER 301 and *Rhr*ER 5439
3. SDS-PAGE gel analysis
4. Determination of the flavin species for *Rhr*ER 2718 by HPLC
5. GC chromatography of all compounds
6. GC-MS analysis of 2-methylcyclopentanone
7. NMR spectra of 2-methyl-N-phebylsuccinimide and levodione

1. Sequence alignment of the three putative ene-reductases from *R. rhodochrous* ATCC 17895 with selected ene-reductases from all 3 OYE classes

```
XenB-I          ------------------------MATIFDPIKLGDLELSNRIIMAPLTRCRA-D---EG   32
PETNR-I         ----------------------MSAEKLFTPLKVGAVTAPNRVFMAPLTRLRSIE---PG   35
MR-I            -----------------MPDTSFSNPGLFTPLQLGSLSLPNRVIMAPLTRSR--T---PD   38
OYE1-II         -----------MSFVKDFKPQALGDTNLFKPIKIGNNELLHRAVIPPLTRMRALH---PG   46
EBPI-II         MTIESTNSFVVPSDTKLIDVTPLGSTKLFQPIKVGNNVLPQRIAYVPTTRFRASK---D-   56
RhrER301        ----------------------MTAPDVLAPAKLGPVTLRNRIIKSATFEGKT-----PD   33
RhrER5439       -------------------MTTAPTPSIFEPARLGPLTLRNRIVKAATFEGVM-----PR   36
YqjM-III        -----------------------MARKLFTPITIKDMTLKNRIVMSPMCMYSSHE---KD   34
TsOYE-III       ------------------------MALLFTPLELGGLRLKNRLAMSPMCQYSATLEGE--   34
OYERo2-III      ------------------------MSVLFEPITFRGVTVPNRVWMAPMCQYSADVTGRDV   36
RhrER2718       ------------------------VSVLFEPLTLRGVTIPNRVWMAPMCQYSADVIGDQV   36
                                           :: *  .      :*

XenB-I          RVPN-ALMAEYYVQRAS--AGLILSEATSVTPMGVGYPDTPGIWSNDQVRGWTNITKAVH   89
PETNR-I         DIPT-PLMGEYYRQRAS--AGLIISEATQISAQAKGYAGAPGLHSPEQIAAWKKITAGVH   92
MR-I            SVPG-RLQQIYYGQRAS--AGLIISEATNISPTARGYVYTPGIWTDAQEAGWKGVVEAVH   95
OYE1-II         NIPNRDWAVEYYTQRAQRPGTMIITEGAFISPQAGGYDNAPGVWSEEQMVEWTKIFNAIH   106
EBPI-II         -HIPSDLQLNYYNARSQYPGTLIITEATFASERGGIDLHVPGIYNDAQAKSWKKINEAIH   115
RhrER301        ALVT-DELIEFHRRPAAGGVGMSTVAYCAVAPEGRTEYGQL-WMRPDALPGLRRLTDAIH   91
RhrER5439       GAVS-DDLINFHAEVARGGAAMTTVAYCAVSPGGRVHRDTL-VMDERALPGLRRLTDAVH   94
YqjM-III        GKLT-PFHMAHYISRAIGQVGLIIVEASAVNPQGRITDQDLGIWSDEHIEGFAKLTEQVK   93
TsOYE-III       --VT-DWHLLHYPTRALGGVGLILVEATAVEPLGRISPYDLGIWSEDHLPGLKELARRIR   91
OYERo2-III      GVPG-DWHRTHLVTRAIGGAGLILTEATAVSPEGRISPADLGIWNDTQTEAFAEINAQLE   95
RhrER2718       GVPN-EWHRTHLVSRAIGGTGLILTEATAVSPEGRISAADLGIWNDTQAQAFAEINAQLA   95
                          .    :     :           .                    :   :

XenB-I          AAGGKIVLQLWHVGRISHPLY-LNGE-----------APVAPSAIQPKGHVSLVRP----   133
PETNR-I         AEDGRIAVQLWHTGRISHSSIQPGGQ-----------APVSASALNANTRTSL-RDENGN   140
MR-I            AKGGRIALQLWHVGRVSHELVQPDGQ-----------QPVAPSALKAEGAECFVEFEDGT   144
OYE1-II         EKKSFVWVQLWVLGWAAFPDNLARDG----------LRYDSASD------NVFMDAEQEA   150
EBPI-II         GNGSFSSVQLWYLGRVANAKDLKDSG----------LPLIAPS-------AVYWDENSEK   158
RhrER301        AEGAAASAQLGHAGPVANQKS-------------TGLPALAPSSS-------F-------   124
RhrER5439       SEGALAAAQIGHAGLVANTLS-------------NKTKTLAPSTR-------L-------   127
YqjM-III        EQGSKIGIQLAHAGRKAE----------------LEGDIFAPSAI-------A-------   123
TsOYE-III       EAGAVPGIQLAHAGRKAGTARPWEGGKP------LGWRVVGPSPI-------P-------   131
OYERo2-III      YFGAVPGIQLGHAGRKGSAHVPWRGGGSLDGDDRLSWQTVAPSAI-------G-------   141
RhrER2718       YFGAVPGIQLAHAGRKASTQVPWRGGKSLPADDRLSWQTVAPSAV-------P-------   141
                   .    *:   *  .                       . *

XenB-I          --LADYPTPRALETAEIAEIV-EAYRTGAENAKAAGFDGVEIHGANGYLLDQFLQSSTNQ   190
PETNR-I         AIRVDTTTPRALELDEIPGIV-NDFRQAVANAREAGFDLVELHSAHGYLLHQFLSPSSNQ   199
MR-I            AGLHPTSTPRALETDEIPGIV-EDYRQAAQRAKRAGFDMVEVHAANACLPNQFLATGTNR   203
OYE1-II         KAKKANNPQHSLTKDEIKQYI-KEYVQAAKNSIAAGADGVEIHSANGYLLNQFLDPHSNT   209
EBPI-II         LAKEAGNELRALTEEEIDHIVEVEYPNAAKHALEAGFDYVEIHGAHGYLLDQFLNLASNK   218
RhrER301        -NPLSMRMIRTATTADITRIA-AAHGSAARLAVEAGFDAVEIHFGHNYFASSFLSPKLNR   182
RhrER5439       -SPPAMGLVKGATLAELDGVV-SDFERAARVAVDAGFDAIEVHLGHNYLLSSFMSPNLNK   185
YqjM-III        -FDEQSATPVEMSAEKVKETV-QEFKQAAARAKEAGFDVIEIHAAHGYLIHEFLSPLSNH   181
TsOYE-III       -FDEGYPVPEPLDEAGMERIL-QAFVEGARRALRAGFQVIELHMAHGYLLSSFLSPLSNQ   189
OYERo2-III      -FGDHTP-PAAATTADIRKVV-ADFAAAAERASRAGFKVVEIHAAHGYLLHQFLSPVSNH   198
RhrER2718       -FGHLAD-PVELTTEGIEKVV-ADFAAAATRALKAEFKVVEIHAAHGYLIHQFLSPESNK   198
                                :       .  ..  :  *  . :*:* .:  :  .*:    *

XenB-I          RTDNYG-GSLENRARLLLEVTDAAIDVWGAGRVGVHLAPRADSHDMGDDN---L------   240
PETNR-I         RTDQYG-GSVENRARLVLEVVDAVCNEWSADRIGIRVSPIGTFQNVDNGPNE--------   250
MR-I            RTDQYG-GSIENRARFPLEVVDAVAEVFGPERVGIRLTPFLELFGLTDD-E--P------   253
OYE1-II         RTDEYG-GSIENRARFTLEVVDALVEAIGHEKVGLRLSPYGVFNSMSGGAE-------TG   262
EBPI-II         RTDKYGCGSIENRARLLLRVVDKLIEVVGANRLALRLSPWASFQGMEIEGE---------   269
RhrER301        RKDSYG-GSLENRARVVLETARSVRDAVG-DKI-----AILAKLNMDDGVP-------GG   228
RhrER5439       RHDRYG-GSVAKRAEYPRRVIEAVRVAAGSSV------AVTAKFNMSDGVP-------KG   231
YqjM-III        RTDEYG-GSPENRYRFLREIIDEVKQVWDG--------PLFVRVSASDYTD-------KG   225
TsOYE-III       RTDAYG-GSLENRMRFPLQVAQAVREVVPREL------PLFVRVSATDWGE-------GG   235
OYERo2-III      RTDEYG-GSFAGRIRLLLEVVDAVRGVWPAEL------PVFVRVSATDWLSEEPGLDADS   251
RhrER2718       RTDRYG-GSFENRIRLLLEILTAVREVWPAEL------PLFVRVSATDWLTEERGLEVDS   251
                * * ** **   * .   .                         .
```

```
XenB-I          --AETFTYVARELGKRG-----IAFICSREKEG---------------------------  266
PETNR-I         --EADALYLIEELAKRG-----IAYLHMSETDLAGG------------------------  279
MR-I            --EAMAFYLAGELDRRG-----LAYLHFNEPDWIGG------------------------  282
OYE1-II         I-VAQYAYVAGELEKRAKAGKRLAFVHLVEPRVTNPFLTEG-------------------  301
EBPI-II         ---EIHSYILQQLQQRADNGQQLAYISLVEPRVTGIYDVS--------------------  306
RhrER301        FWVDEAIQVAQWLEASGSV--DALELTMGSSLLNPMYLFKGDAPIREFAAAMPQPVRLGV  286
RhrER5439       LWLDQSLPIAQILEADGHL--DAMQLTGGSSLLNGMYFFRGEVPLAEFVASQPKLVGYGL  289
YqjM-III        LDIADHIGFAKWMKEQGV---DLIDCSSG-------------------------------  251
TsOYE-III       WSLEDTLAFARRLKELGV---DLLDCSSG-------------------------------  261
OYERo2-III      WTPDQTVSLVQALADLGV---DLVDVSSG-------------------------------  277
RhrER2718       WTADQTVALANILSDYGV---DLVDVSTG-------------------------------  277
                        .   :   .

XenB-I          -----------------ADSLGPQLKEAFGGAYIANERFTKDSANAW--L--AEGKADAV  305
PETNR-I         --------------KPYSEAFRQKVRERFHGVIIGAGAYTAEKAEDL--I--GKGLIDAV  321
MR-I            ---------D----ITYPEGFREQMRQRFKGGLIYCGNYDAGRAQAR--L--DDNTADAV  325
OYE1-II         ---------E----GEYEGGSNDFVYSIWKGPVIRAGNFALHPEV---VREEVKDKRTLI  345
EBPI-II         ---------L----KDQQGRSNEFAYKIWKGNFIRAGNYTYDAPEFKTLINDLKNDRSII  353
RhrER301        QLVGKSMLHAYPYKPLFMLEEARQIRAAVKLPLVLLGGVTD-KAGMDTAM--AEGF-EFV  342
RhrER5439       KFYGPKLFPTYPFEEGFFLPFARQFRQALRMPLILLGGINR-VDTIEHAL--DEGF-EFV  345
YqjM-III        ---ALV-HADINVFPGYQVSFAEKIREQADMATGAVGMITD-GSMAEEIL--QNGRADLI  304
TsOYE-III       ---GVVLRVRIPLAPGFQVPFADAVRKRVGLRTGAVGLITT-PEQAETLL--QAGSADLV  315
OYERo2-III      ---GVA-SARIPIGPGYQVPFARRIQNETTVPAAAVGLITE-PEQAERIV--ESGEAVAV  330
RhrER2718       ---GNSPAAQIPVEPGYQVPFARRLQNESLLPAAAVGLITE-PEQAEKIV--EDGSAVAV  331
                                                      .    :

XenB-I          AFGVPFIANPDLPARLKADAP-------LNEPRPELFYGKGPVGYIDYPTL---------  349
PETNR-I         AFGRDYIANPDLVARLQKKAE-------LNPQRPESFYGGGAEGYTDYPSL---------  365
MR-I            AFGRPFIANPDLPERFRLGAA-------LNEPDPSTFYGGAEVGYTDYPFLDNGHDRLG-  377
OYE1-II         GYGRFFISNPDLVDRLEKGLP-------LNKYDRDTFYQMSAHGYIDYPTYEEALK-LGW  397
EBPI-II         GFSRFFTSNPDLVEKLKLGKP-------LNYYNREEFYKYYNYGYNSYDESEKQVI--GK  404
RhrER301        AMARALLREPDLINRIAAESDTQSLCIHCNKCMPTIFSGARCVLVERV------------  390
RhrER5439       AMARALLRDPQLVNKFQAESVDEGLCIHCNKCMPTIYTGTRCVVRDAL-VVREAPR----  400
YqjM-III        FIGRELLRDPFFARTAAKQLNTE-----IP--APVQYERGW-------------------  338
TsOYE-III       LLGRVLLRDPYFPLRAAKAL--G-----VAPEVPPQYQRGF-------------------  349
OYERo2-III      FLGRELLRDPYWPRKAALVLNAQ-----VTPQIPAQYARAY-------------------  366
RhrER2718       LLGRELLRDPYWARRAARELNAE-----VGPHIPSQYARAF-------------------  367
                  .     :*                          :

XenB-I          --- 349
PETNR-I         --- 365
MR-I            --- 377
OYE1-II         DKK 400
EBPI-II         PLA 407
RhrER301        --- 390
RhrER5439       PTQ 403
YqjM-III        --- 338
TsOYE-III       --- 349
OYERo2-III      --- 366
RhrER2718       --- 367
```

Figure S1. Amino acid sequence alignment of the three ene-reductases from *R. rhodochrous* ATCC 17895 with OYEs from all 3 classes (Scholtissek et al. 2017). The sequence of *Pseudomonas fluorescens* I-C (XenB), *Enterobacter cloacae* PB2 (PETNR), *P. putida* M10 (MR), *Saccharomyces pastorianus* (OYE1), *Candida albicans* (EBP1), *Bacillus subtilis* strain 168 (YqjM), *Thermus scotoductus* SA-01 (TsOYE) AND *Rhodococcus opacus* 1CP (OYERo2) are compared with the protein sequence of the ene-reductases from *R. rhodochrous* ATCC 17895 (*Rhr*ER301, *Rhr*ER2718 and *Rhr*ER5439). The alignment was created using the online available Clustal Omega alignment tool (Sievers et al. 2011). The amino acid residues involved in substrate binding (*black frames*), FMN binding (*grey shades*), and the catalytic sites (*white letters in black*) are indicated.

2. Solubilisation of *Rhr*ER 301 and *Rhr*ER 5439

The expression of *Rhr*ER 2718 gave large amounts of soluble protein when expressed overnight from pET28 at 30°C. Unfortunately, both *Rhr*ER 301 and *Rhr*ER 5439 resulted in no visible soluble protein under the same conditions. In order to improve this several parameters were changed as shown in Table S1. Unfortunately, all these attempts did not result in soluble protein, so we did not pursue this any further. The reason it did not express in soluble form might be due to the fact that *Rhodococcus* is a Gram-positive organism, while *E. coli* is Gram-negative, which may give rise to different internal conditions of the cell. Another option is that these enzymes are also redundant in *Rhodococcus* and are either never expressed, or only expressed under certain (stress) conditions which we did not mimic in our attempts.

Table S1 Different strategies to improve soluble expression of *Rhr*ER 301 and *Rhr*ER 5439

**Attempt**	**Modification**	**Reason**
1	Combinations of different temperatures, IPTG concentrations and incubation times	Slower expression could lead to more soluble enzyme
2	Co-expression with all different chaperones from the TAKARA chaperone plasmid set at different temperatures	Chaperones can help with protein folding
3	Expression in *E. coli* BL21 (DE3) Rosetta2 cells	Rosetta2 contains tRNAs for rare codons in *E. coli*
4	Expression in pBAD-His plasmid at different arabinose concentrations and temperatures	Better control of expression level using arabinose
5	Resolubilisation of inclusion bodies	Trying to resolubilise the large amount of protein present in insoluble fraction
6	Expression without His-tag	His-tag might impair proper folding
7	Expression in low copy plasmid	Less expression can improve folding

3. SDS-PAGE gel analysis

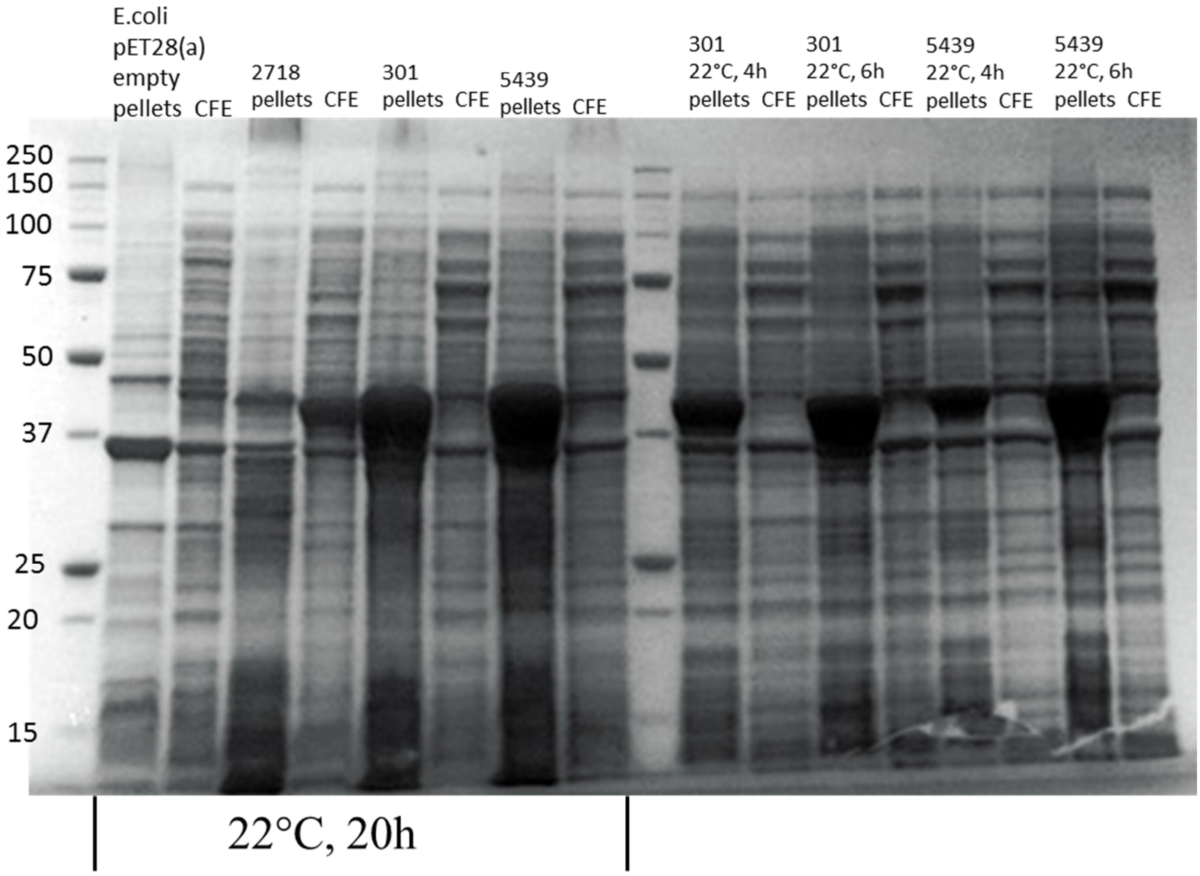

Figure S2 SDS-PAGE gel analysis of different growing conditions for the three putative ene-reductases *Rhr*ER 301, *Rhr*ER 2718 and *Rhr*ER 5439. The gel revealed the optimal conditions for culturing.

4. Determination of the flavin species for *Rhr*ER 2718 by HPLC

The pure enzyme *Rhr*ER 2718 was denatured by heat and the protein was removed by centrifugation and a Microcon YM3 (Millipore, MWCO, 3000 Da) centrifugal concentrator device. The resulting sample was analyzed by HPLC to identify the flavin cofactor. A reverse phase C18 HPLC column connected to a Shimadzu LC10Ai HPLC system was used. Ammonium acetate (50 mM, pH 6.0) and 70% acetonitrile in ammonium acetate (50 mM, pH 6.0) were used as mobile phase, the retention times of FAD and FMN are 6.53 and 9.12 min, respectively as shown below.

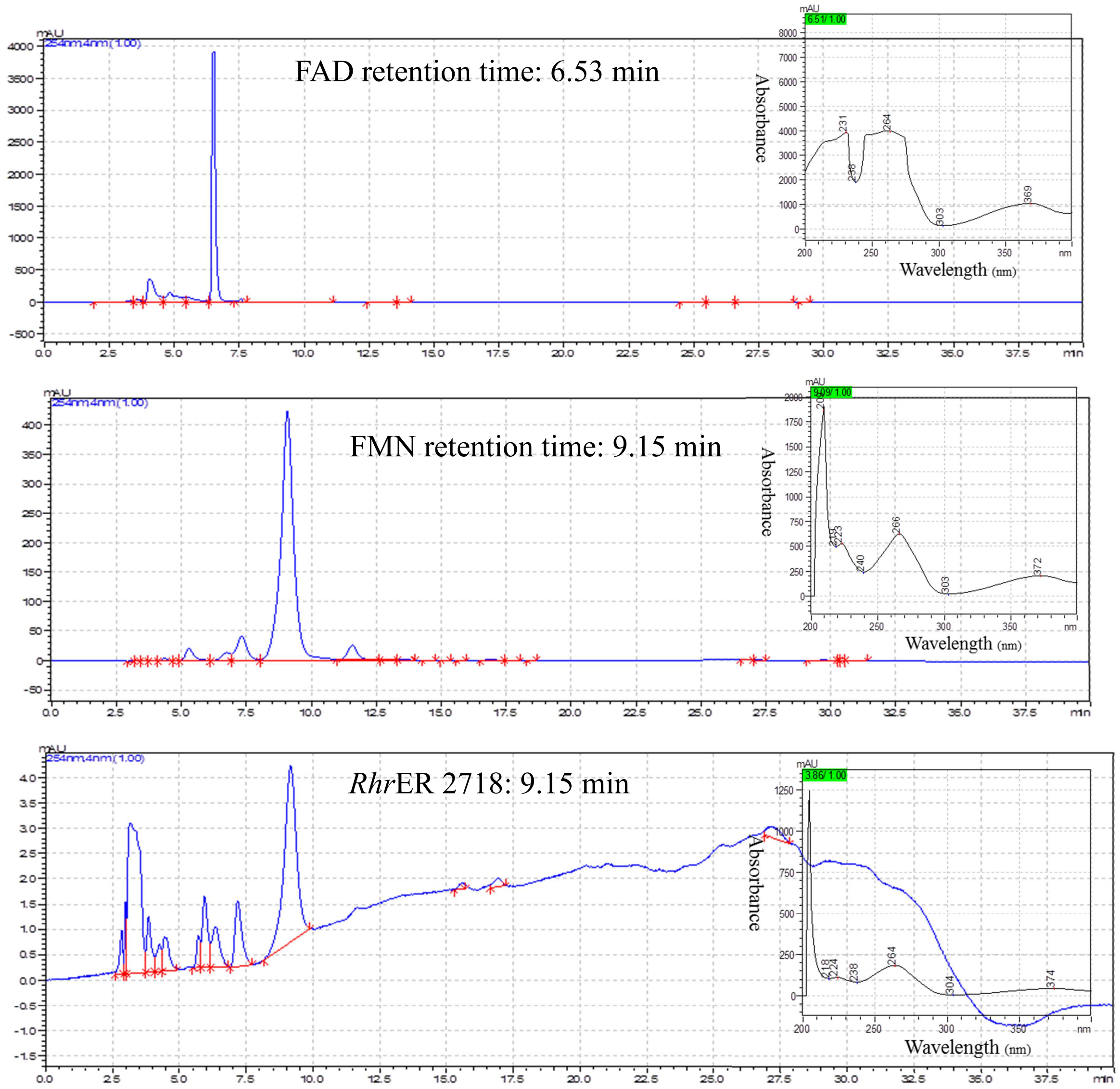

Figure S3 HPLC chromatogram of FAD and FMN standards and supernatant from denatured *Rhr*ER 2718.

5. GC chromatography of all compounds

5.1 GC analysis

A: Column CP sil 5 CB (25 m x 0.25 mm x 1.2 µm)
B: Column HYDRODEX β-TBDM (50 m x 0.4 mm x 0.25 µm)
C: Column LIPODEX E (50 m x 0.25 mm x 0.25 µm)
D: Column CP Chirasil-Dex CB (25 m x 0.32 mm x 0.25 µm)

Table S2 GC programs and retention times for the determination of conversion, yield and *ee*

Column	Program*	Compounds	Retention time (min)
**A**	110/8/30/220/1/30/350/1	2-cyclopentenone<br>cyclopentanone<br>cyclopentanol	4.3<br>3.7<br>3.5
**A**	125/7.5/25/195/1/30/350/1	2-cyclohexenone<br>cyclohexanone<br>cyclohexanol	5.0<br>4.4<br>4.1
**A**	130/8.5/25/185/1/30/350/1	2-cycloheptenone<br>cycloheptanone	7.5<br>6.4
**A**	120/3/25/150/7/30/350/1	4,4-dimethylcyclohexenone<br>4,4-dimethylcyclohexanone	6.3<br>6.1
**B**	110/9/25/170/1/25/250/1	2-methyl-2-cyclopentenone<br>2-methylcyclopentanone	7.8<br>(*R*) 6.3, (*S*) 6.1
**C**	100/4/5/110/4/25/220/1	2-methyl-2-cyclohexenone<br>2-methylcyclohexanone	8.3<br>(*R*) 6.3, (*S*) 6.1
**A**	95/8/25/215/1/30/350/1	trans-2-hexenal<br>hexanal<br>trans-2-hexenol	6.4<br>4.8<br>6.6
**A**	120/3/25/190/5/30/350/1	1-nitro-1-cyclohexene<br>1-nitrocyclohexane	7.8<br>6.6
**D1**	120/10.8/25/225/1	ketoisophorone<br>levodione	7.6<br>(*R*) 8.8, (*S*) 9.4
**D2**	120/3/10/150/4/15/225/1	ketoisophorone<br>levodione	7.1<br>(*R*) 7.7, (*S*) 7.9
**B**	115/3.5/15/180/23.5/15/250/1	2-methyl-*N*-phenylmaleimide<br>2-methyl-*N*-phenylsuccinimide	22.3<br>(*R*)30.0 , (*S*)29.4

* Program: initial temperature (°C) / time (min) / slope (°C/min) / temperature (°C) / time (min) / slope (°C/min) / temperature (°C) / time (min)

5.2- 5.13 Calibration standards and reaction for all substrates and possible products

5.2. Figure S4: 2-cyclopentenone, 2-cyclopentanone, 2-cyclopentanol and dodecane (IS)

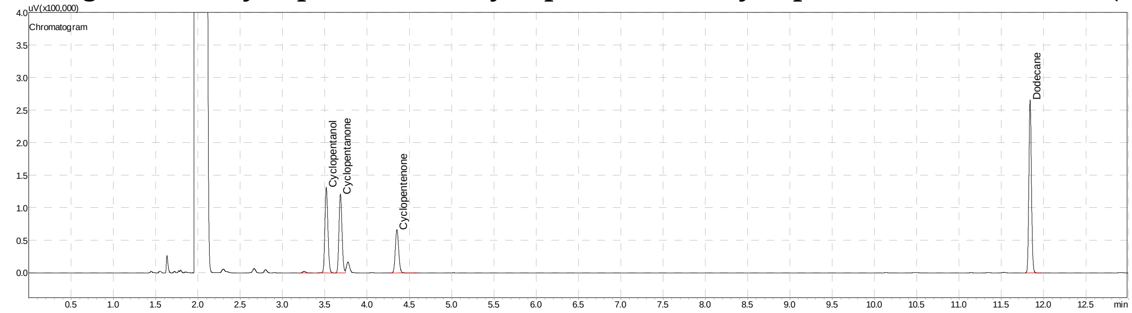

Reaction of Rhr*ER 2718 with 2-cyclopentenone*

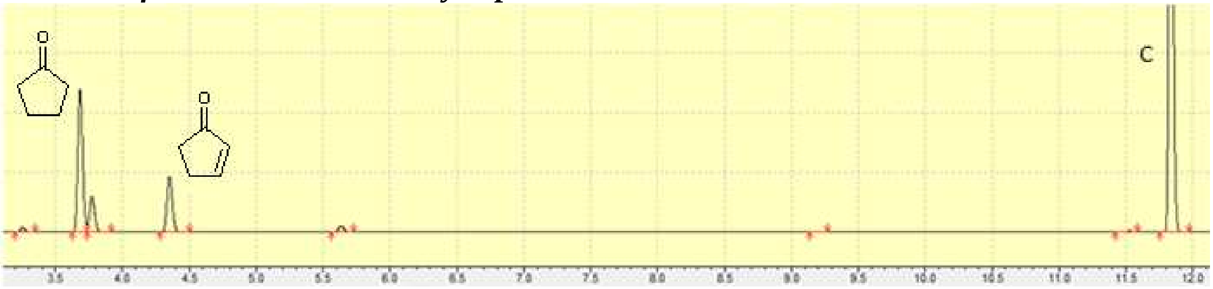

5.3. Figure S5: 2-cyclohexenone, 2-cyclohexanone, 2-cyclohexanol and dodecane (IS)

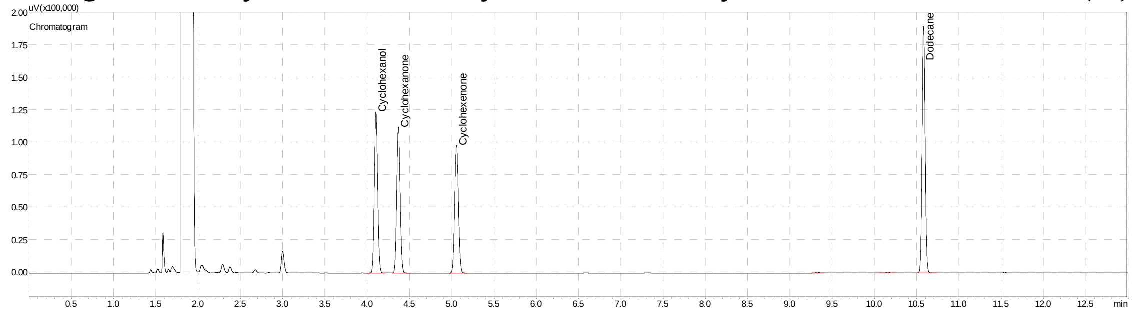

Reaction of Rhr*ER 2718 with 2-cyclohexenone*

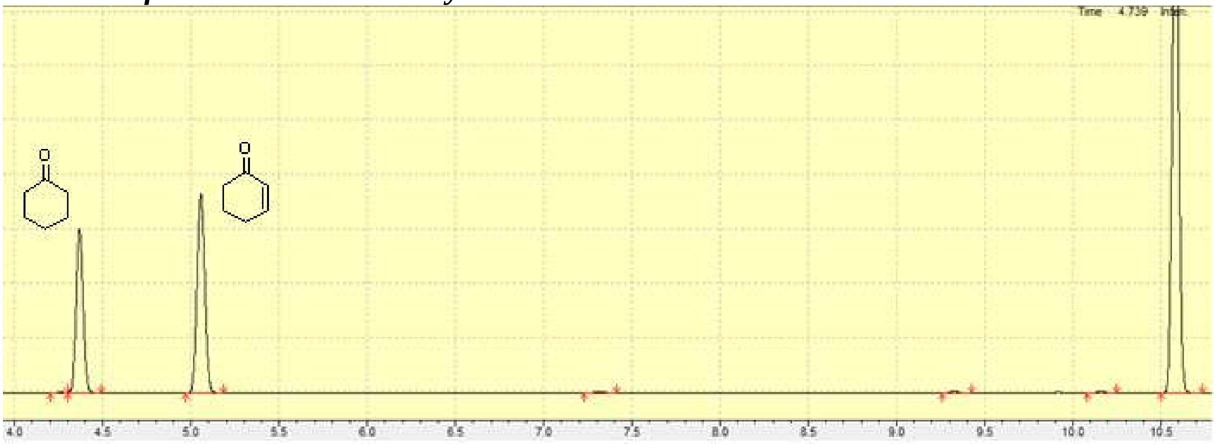

5.4. Figure S6: 2-cycloheptenone, 2-cycloheptanone and dodecane (IS)

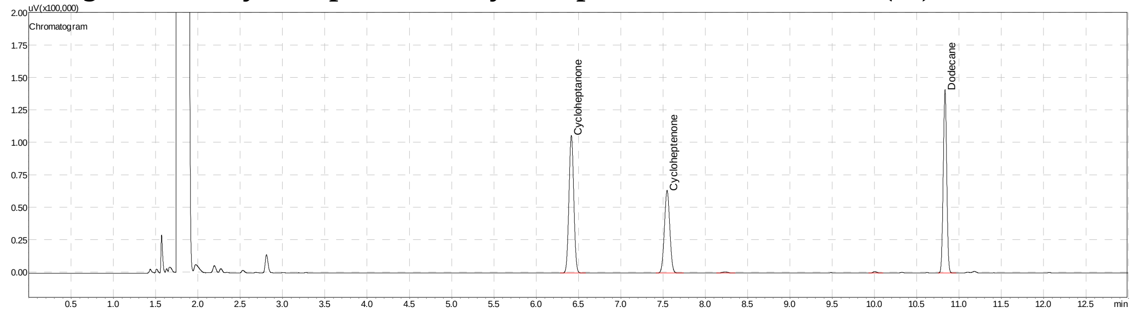

Reaction of Rhr*ER 2718 with 2-cycloheptenone*

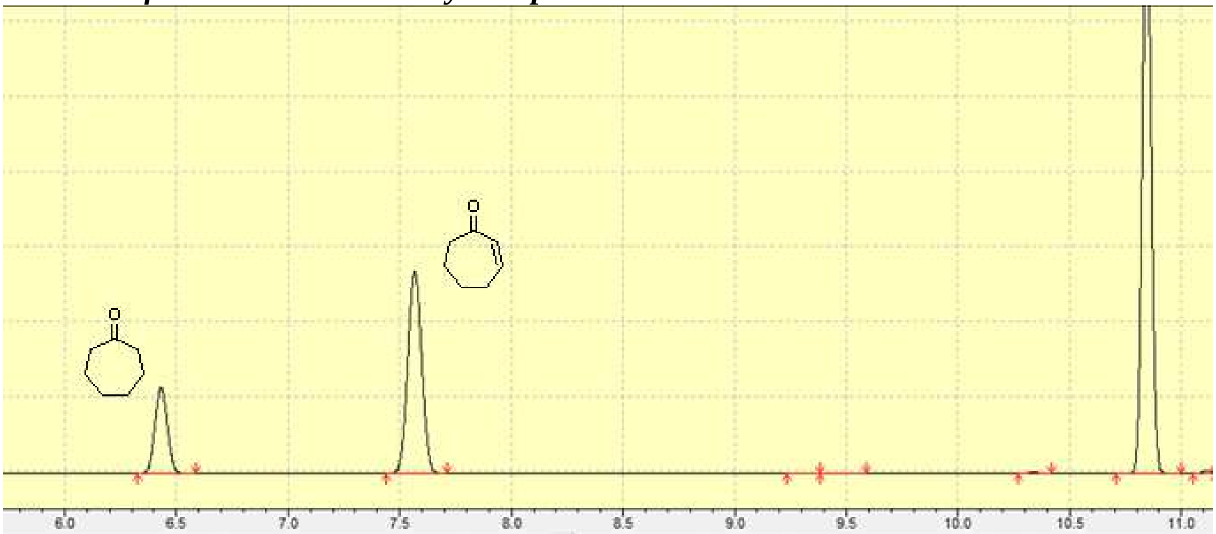

5.5. Figure S7: 4,4-dimethylcyclohexenone, 4,4-dimethylcyclohexanone and dodecane (IS)

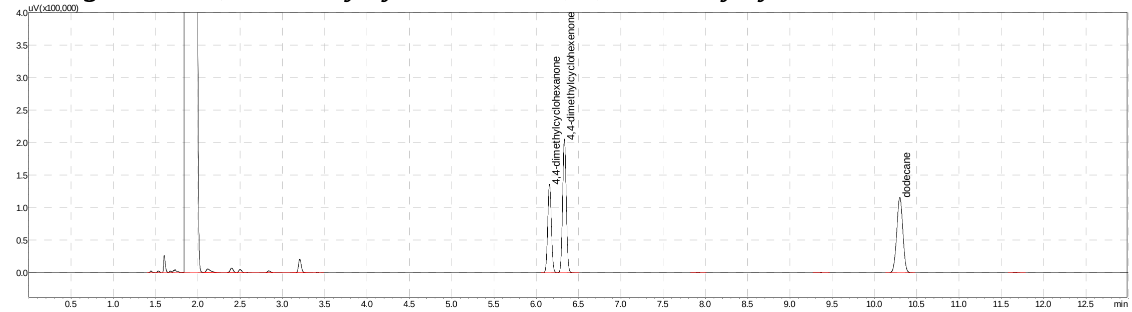

Reaction of Rhr*ER 2718 with 4,4-dimethylcyclohexenone*

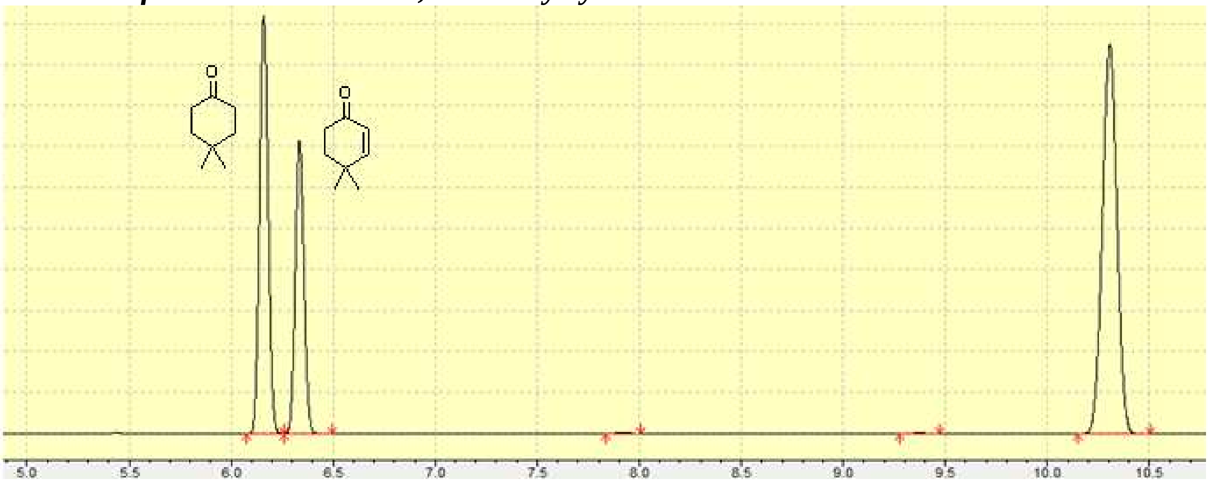

5.6. Figure S8: 1-nitro-1-cyclohexene, nitrocyclohexane and dodecane (IS)

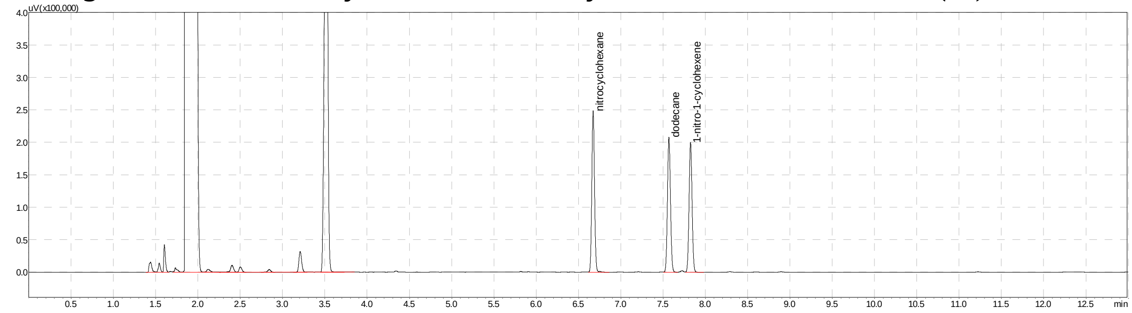

Reaction of RhrER 2718 with 1-nitro-1-cyclohexene

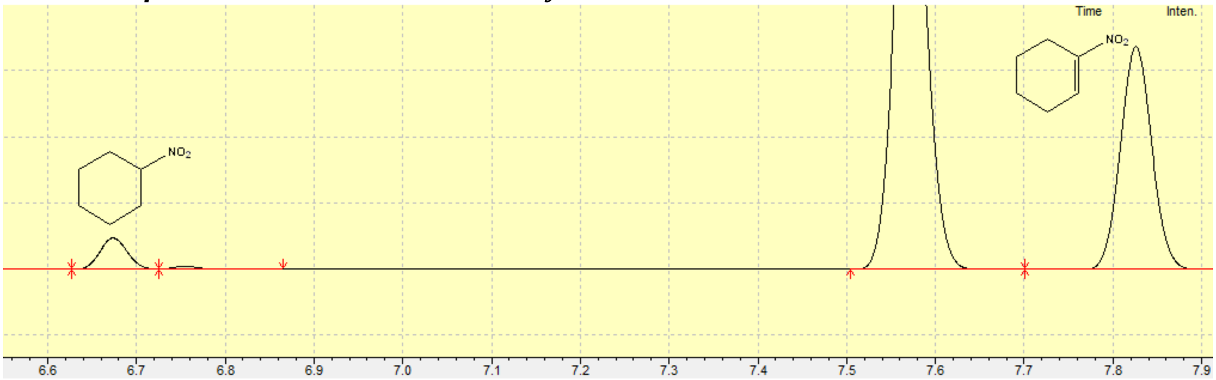

5.7. Figure S9: trans-2-hexenal, hexanal, trans-2-hexenol and dodecane (IS)

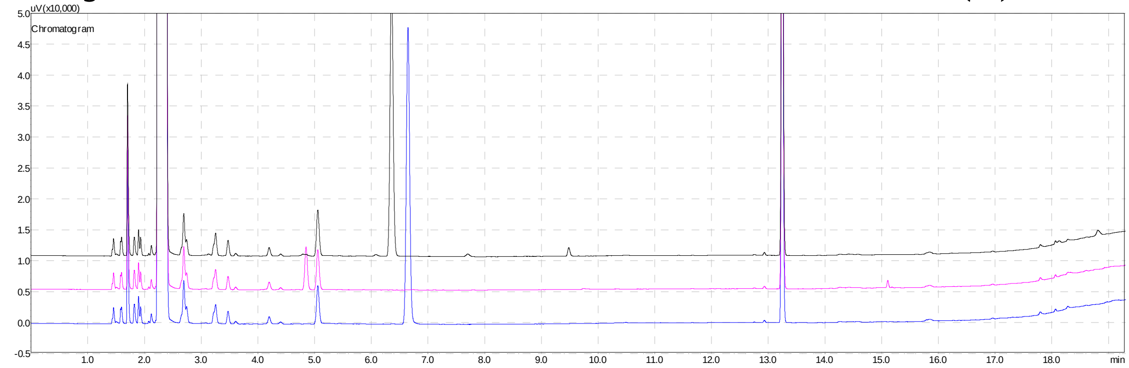

black = trans-2-hexenal (6.4), pink = hexanal (4.8), blue = trans-2-hexenol (6.6), dodecane (13.2)

Reaction of RhrER 2718 with trans-2-hexenal

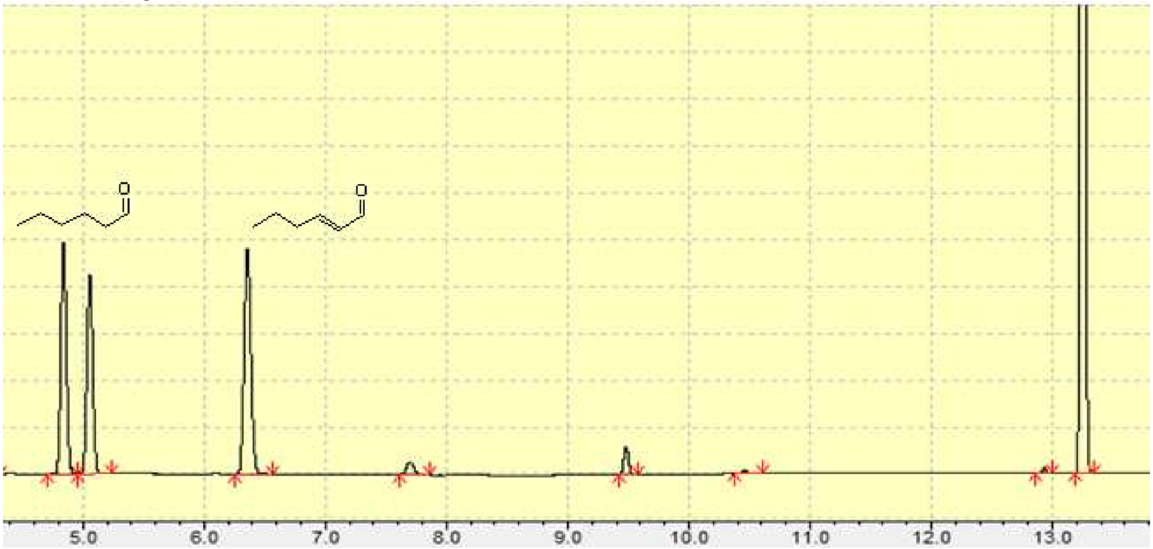

5.8. Figure S10: 2-methyl-N-phenylmaleimide, (2S)- and (2R)-methyl-N-phenylsuccinimide and dodecane (IS)

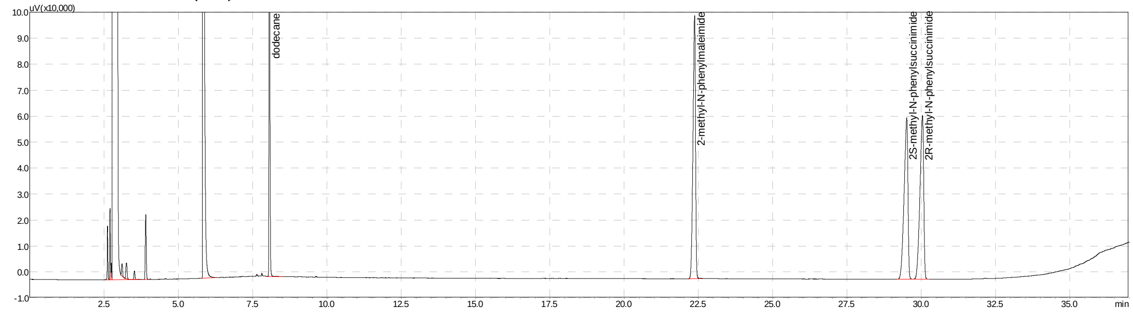

Reaction of Rhr*ER 2718 with 2-methyl-N-phenylmaleimide*

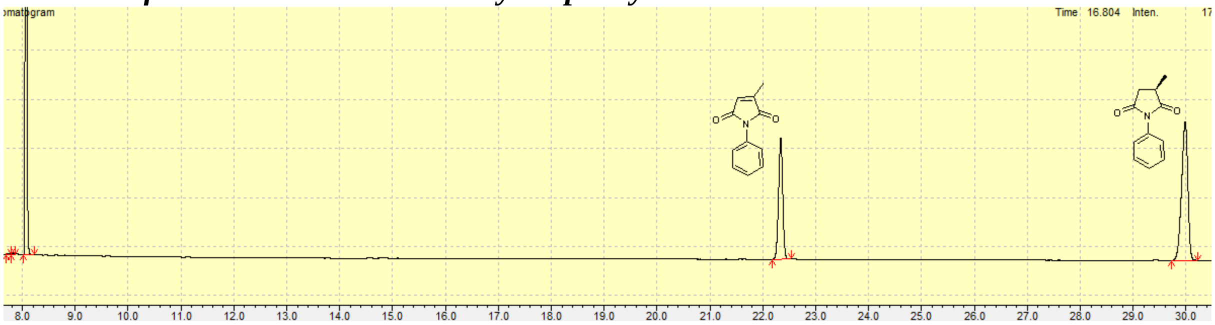

5.9. Figure S11: 2-methylcyclopentenone, (2S)- and (2R)-methylcyclopentanone and dodecane (IS)

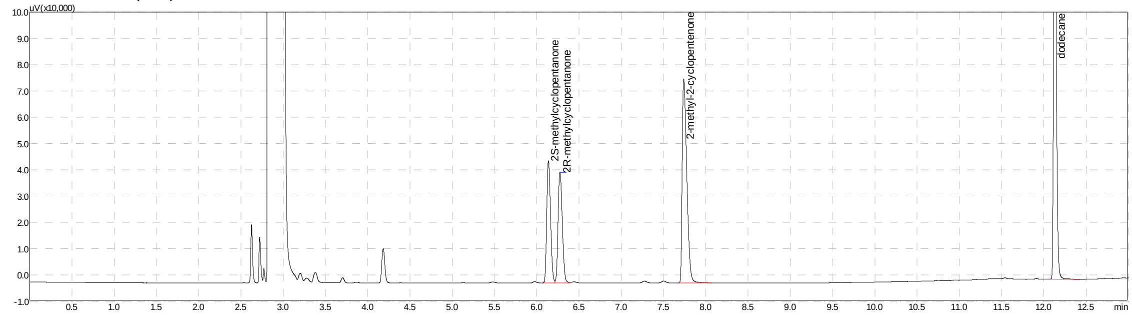

Reaction of Rhr*ER 2718 with 2-methylcyclopentenone*

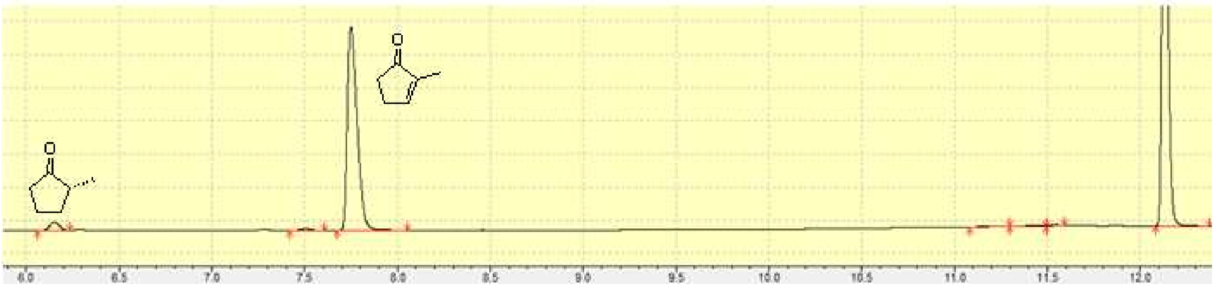

5.10. Figure S12: 2-methylcyclohexenone, (2S)- and (2R)-methylcyclohexanone and dodecane (IS)

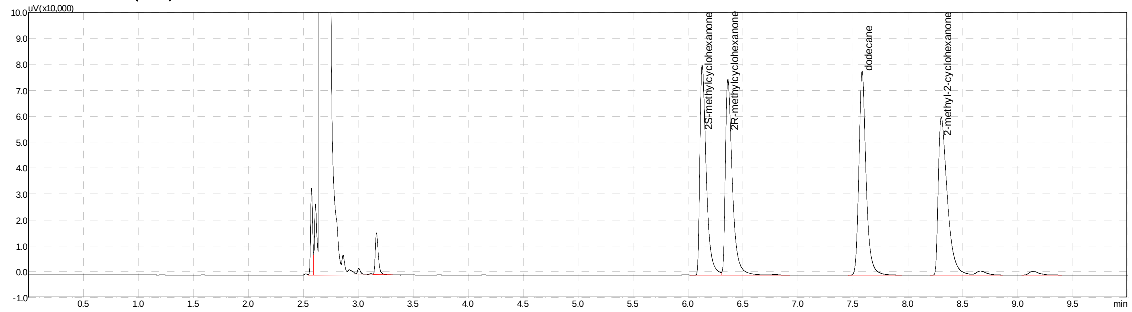

Reaction of* Rhr*ER 2718 with 2-methylcyclohexenone

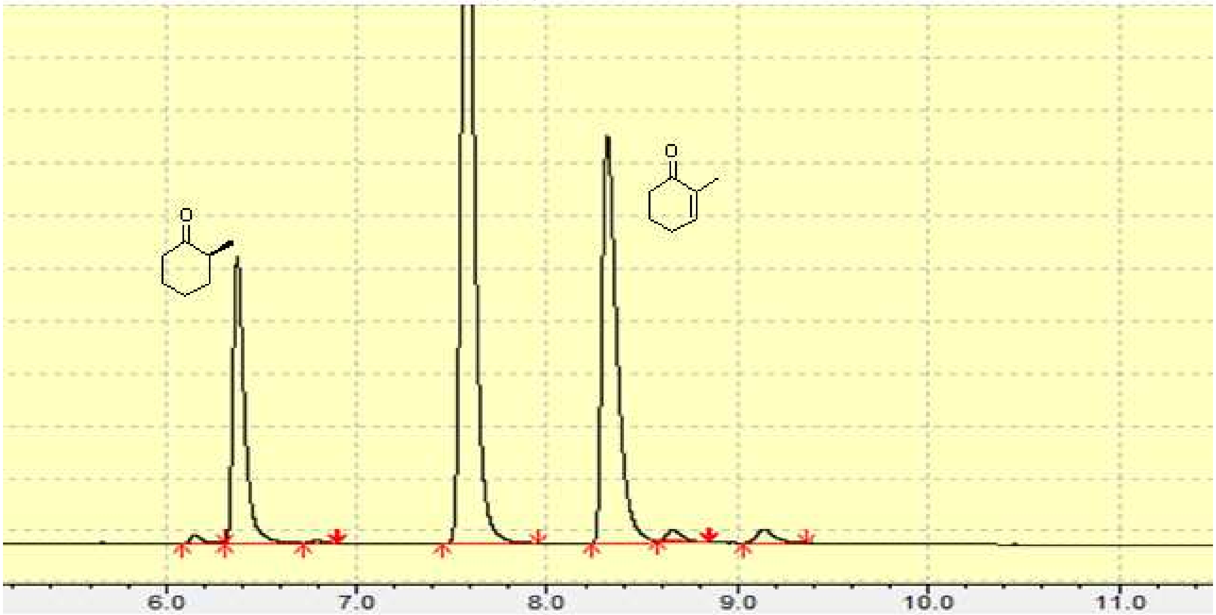

5.11. Figure S13: ketoisophorone, (R)-levodione and dodecane (IS) on column D1

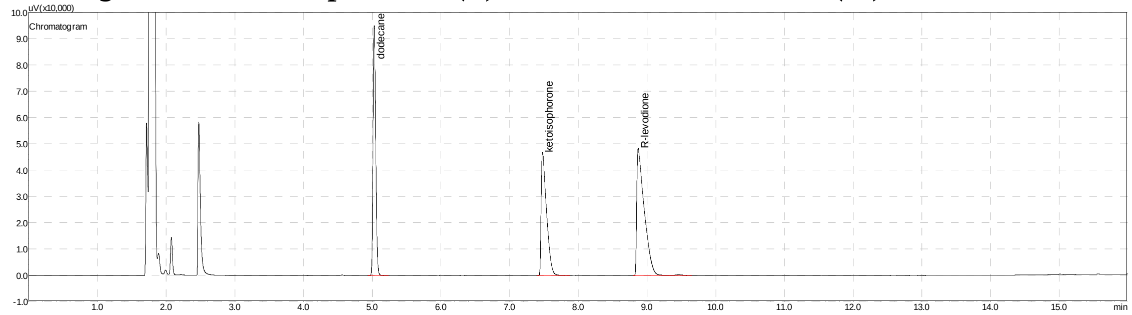

Reaction of RhrER *2718 with ketoisophorone*

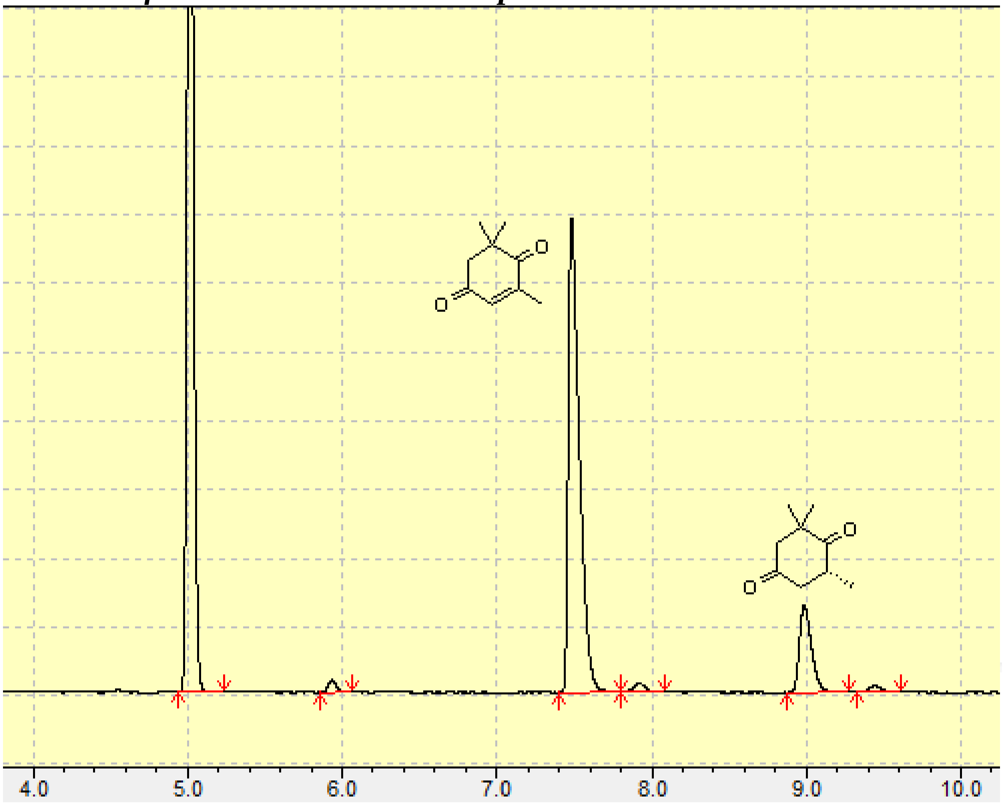

5.12. Figure S14: (R)- and (S)- levodione on column D2

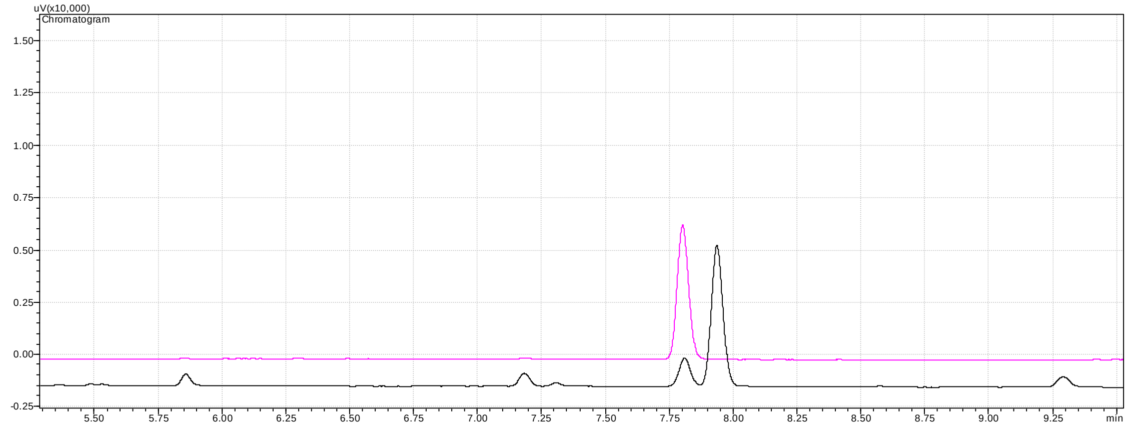

(*R*)-levodione (pink); (*S*)-levodione (black, racemises relatively quickly in phosphate buffer (pH 7.0)).

6. Figure S15: GC-MS analysis of 2-methylcyclopentanone

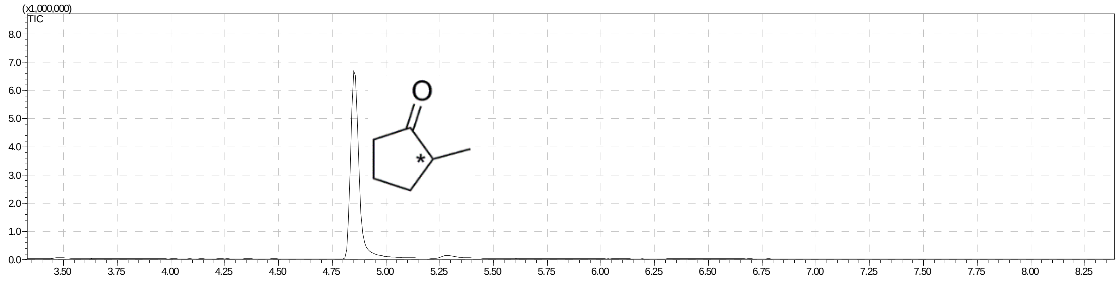

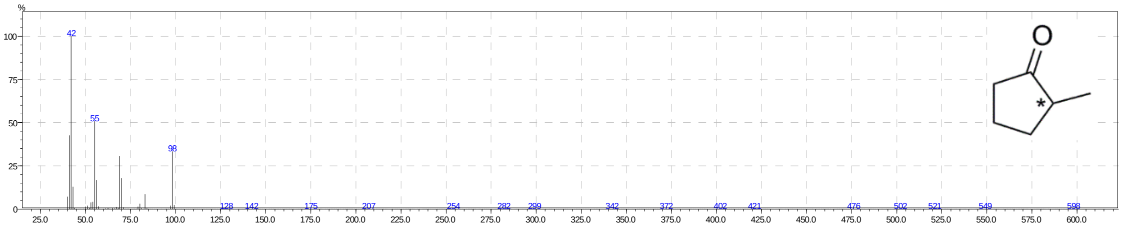

7. NMR spectra

All spectra were according to spectra reported before (Fryszkowska et al. 2009; Hall et al. 2007).

7.1 Figure S16: ^{1}H NMR spectrum of 2-methyl-*N*- phenylsuccinimide ($CDCl_3$, 400 MHz)

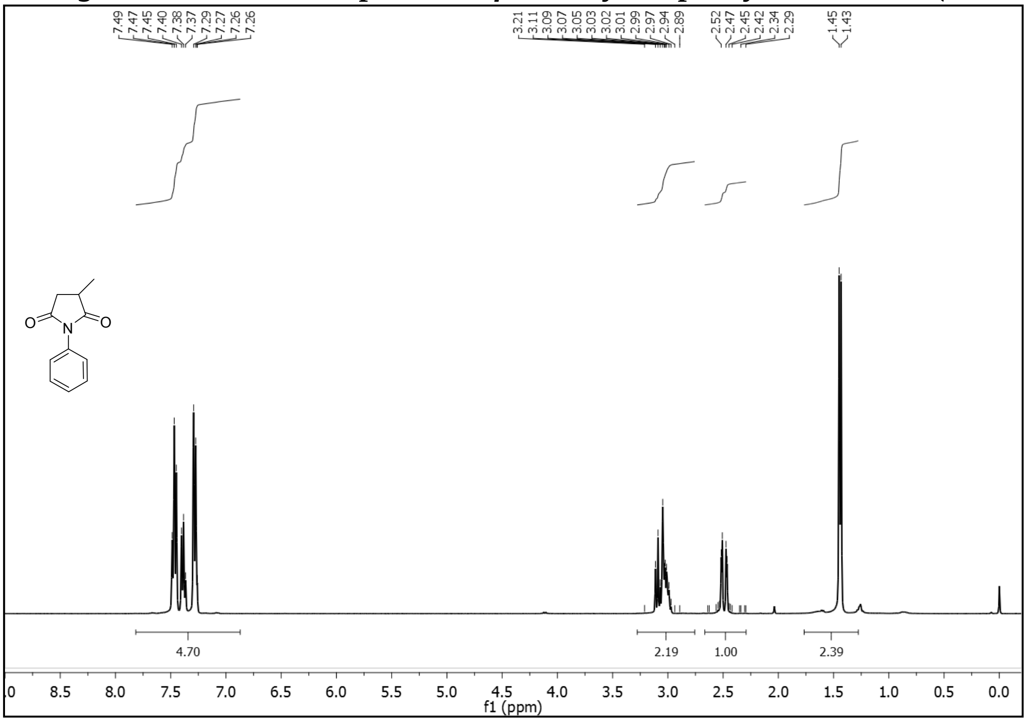

7.2 Figure S17: ^{13}C NMR spectrum of 2-methyl-*N*- phenylsuccinimide ($CDCl_3$, 100 MHz)

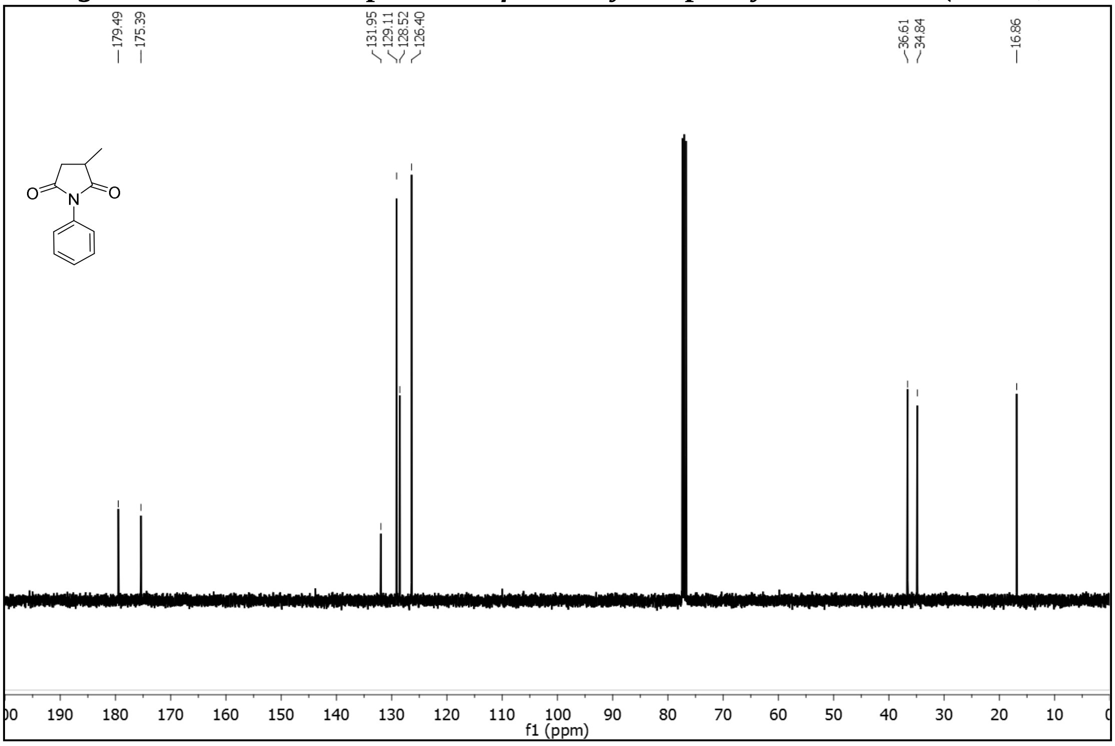

7.3Figure S18: ^{1}H NMR spectrum of levodione: ^{1}H NMR ($CDCl_3$, 400 MHz)

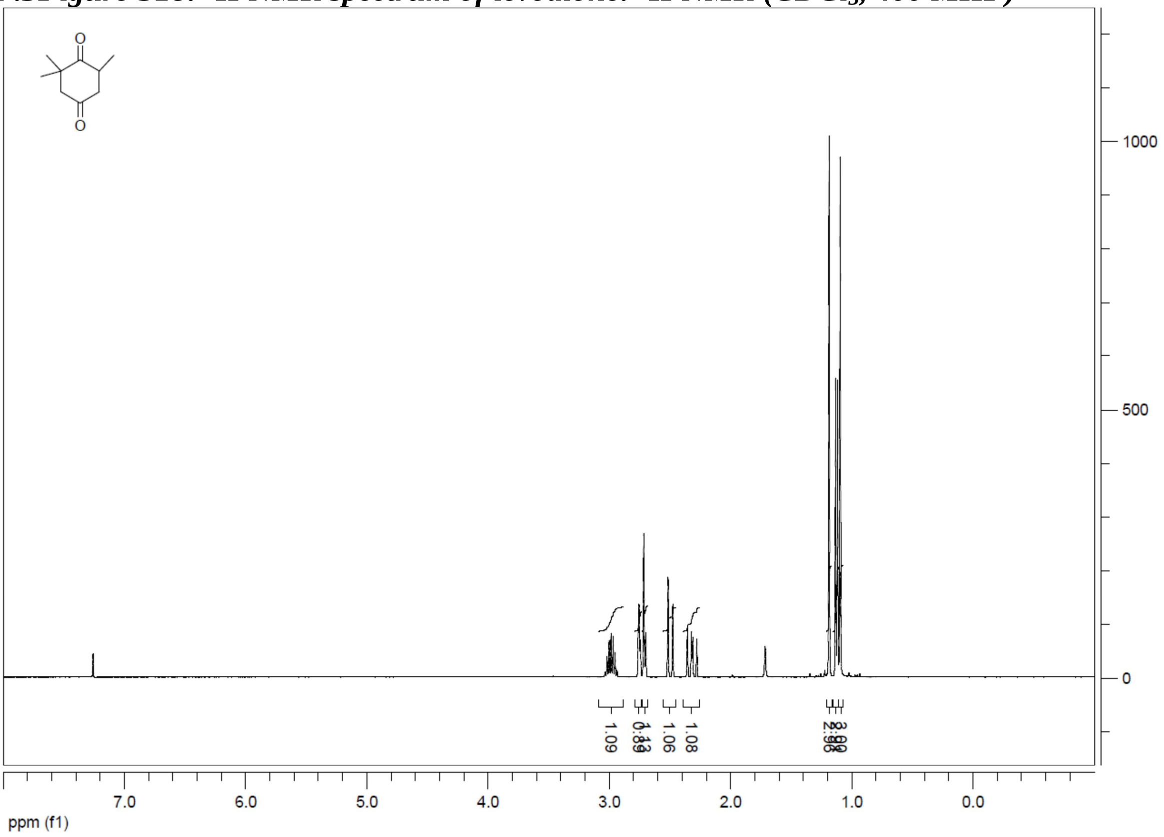

7.4Figure S19: ^{13}C NMR spectrum of levodione: ^{1}H NMR ($CDCl_3$, 100 MHz)

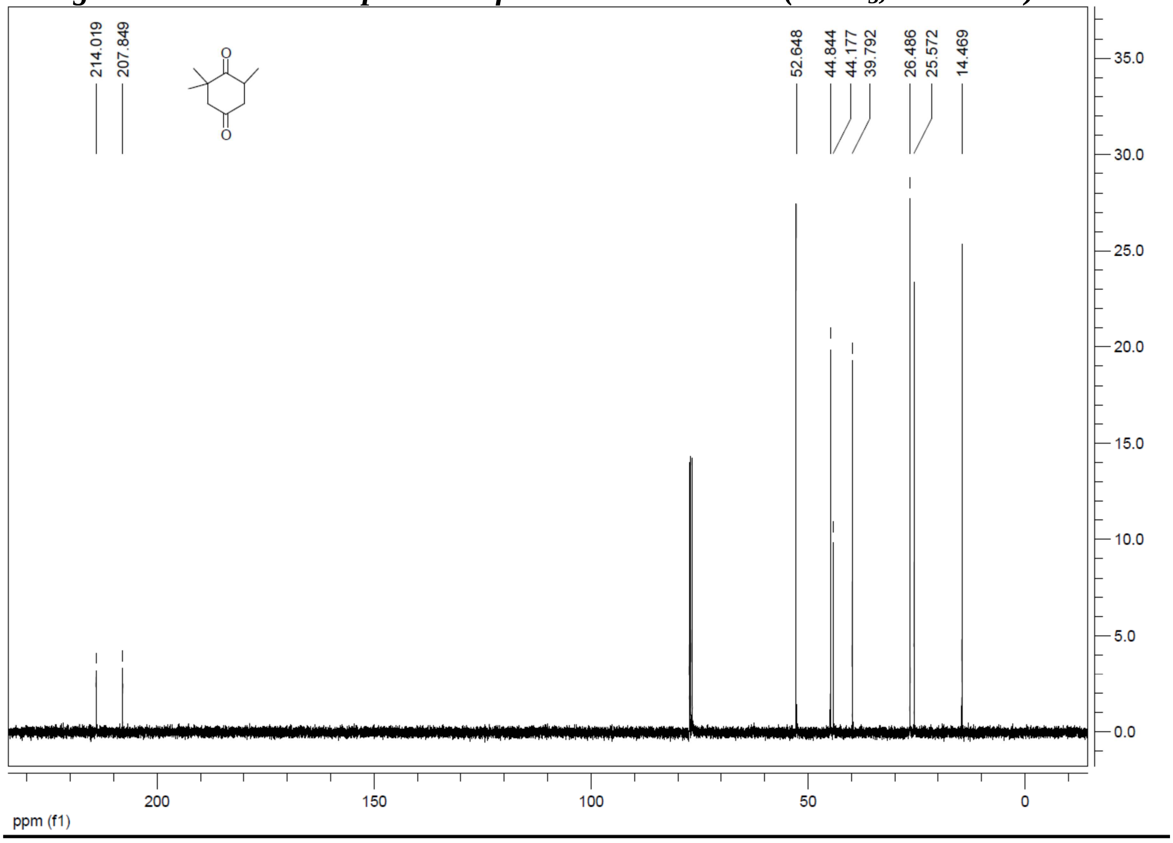

References

Fryszkowska A, Toogood H, Sakuma M, Gardiner JM, Stephens GM, Scrutton NS (2009) Asymmetric reduction of activated alkenes by pentaerythritol tetranitrate reductase: Specificity and control of stereochemical outcome by reaction optimisation. Advanced Synthesis & Catalysis 351(17):2976-2990 doi:10.1002/adsc.200900574

Hall M, Stueckler C, Kroutil W, Macheroux P, Faber K (2007) Asymmetric bioreduction of activated alkenes using cloned 12-oxophytodienoate reductase isoenzymes OPR-1 and OPR-3 from *Lycopersicon esculentum* (tomato): a striking change of stereoselectivity. Angewandte Chemie International Edition 46(21):3934-3937 doi:10.1002/anie.200605168

Scholtissek A, Tischler D, Westphal A, van Berkel W, Paul C (2017) Old Yellow Enzyme-catalysed asymmetric hydrogenation: Linking family roots with improved catalysis. Catalysts 7(5):130 doi:10.3390/catal7050130

Sievers F, Wilm A, Dineen D, Gibson TJ, Karplus K, Li W, Lopez R, McWilliam H, Remmert M, Söding J, Thompson JD, Higgins DG (2011) Fast, scalable generation of high-quality protein multiple sequence alignments using Clustal Omega. Molecular Systems Biology 7(1) doi:10.1038/msb.2011.75
